# Adenosine A_2A_ receptor as a potential regulator of *Mycobacterium leprae* survival mechanisms: new insights into leprosy neural damage

**DOI:** 10.3389/fphar.2024.1399363

**Published:** 2024-06-28

**Authors:** Plinio Marcos Freire dos Santos, Chyntia Carolina Díaz Acosta, Thabatta Leal Silveira Andrezo Rosa, Michelle Harumi Ishiba, André Alves Dias, Antonio Marcos Rodrigues Pereira, Luísa Domingos Gutierres, Melissa Pontes Pereira, Matheus da Silva Rocha, Patrícia Sammarco Rosa, Daniele F. F. Bertoluci, José Roberto Meyer-Fernandes, Fabricio da Mota Ramalho Costa, Maria Angela M. Marques, John T. Belisle, Roberta Olmo Pinheiro, Luciana Silva Rodrigues, Maria Cristina Vidal Pessolani, Marcia Berrêdo-Pinho

**Affiliations:** ^1^ Laboratório de Microbiologia Celular, Instituto Oswaldo Cruz, Fundação Oswaldo Cruz, Rio de Janeiro, Brazil; ^2^ Departamento de Biología Molecular y Biotecnología, Instituto de Investigaciones en Ciencias de la Salud, Universidad Nacional de Asunción, San Lorenzo, Paraguay; ^3^ Divisão de Pesquisa e Ensino, Instituto Lauro de Souza Lima, São Paulo, Brazil; ^4^ Departamento de Doenças Tropicais, Faculdade de Medicina de Botucatu, Universidade Estadual Paulista, Botucatu, Brazil; ^5^ Laboratório de Bioquímica Celular, Instituto de Bioquímica Médica Leopoldo de Meis, Centro de Ciências da Saúde, Universidade Federal do Rio de Janeiro, Rio de Janeiro, Brazil; ^6^ Department of Microbiology, Immunology and Pathology, Colorado State University, Fort Collins, CO, United States; ^7^ Laboratório de Hanseníase, Instituto Oswaldo Cruz, Fundação Oswaldo Cruz, Rio de Janeiro, Brazil; ^8^ Laboratório de Imunopatologia, Faculdade de Ciências Médicas, Universidade do Estado do Rio de Janeiro, Rio de Janeiro, Brazil

**Keywords:** Leprosy, schwann cell, *M. leprae*, adenosinergic system, A_2A_ receptor, lipid droplet, bacterial viability

## Abstract

**Background:**

Leprosy is a chronic infectious disease caused by *Mycobacterium leprae*, which can lead to a disabling neurodegenerative condition. *M. leprae* preferentially infects skin macrophages and Schwann cells–glial cells of the peripheral nervous system. The infection modifies the host cell lipid metabolism, subverting it in favor of the formation of cholesterol-rich lipid droplets (LD) that are essential for bacterial survival. Although researchers have made progress in understanding leprosy pathogenesis, many aspects of the molecular and cellular mechanisms of host–pathogen interaction still require clarification. The purinergic system utilizes extracellular ATP and adenosine as critical signaling molecules and plays several roles in pathophysiological processes. Furthermore, nucleoside surface receptors such as the adenosine receptor A_2A_R involved in neuroimmune response, lipid metabolism, and neuron–glia interaction are targets for the treatment of different diseases. Despite the importance of this system, nothing has been described about its role in leprosy, particularly adenosinergic signaling (AdoS) during *M. leprae*–Schwann cell interaction.

**Methods:**

*M. leprae* was purified from the hind footpad of athymic *nu/nu* mice. ST88-14 human cells were infected with *M. leprae* in the presence or absence of specific agonists or antagonists of AdoS. Enzymatic activity assays, fluorescence microscopy, Western blotting, and RT-qPCR analysis were performed. *M. leprae* viability was investigated by RT-qPCR, and cytokines were evaluated by enzyme-linked immunosorbent assay.

**Results:**

We demonstrated that *M. leprae*-infected Schwann cells upregulated CD73 and ADA and downregulated A_2A_R expression and the phosphorylation of the transcription factor CREB (p-CREB). On the other hand, activation of A_2A_R with its selective agonist, CGS21680, resulted in: 1) reduced lipid droplets accumulation and pro-lipogenic gene expression; 2) reduced production of IL-6 and IL-8; 3) reduced intracellular *M. leprae* viability; 4) increased levels of p-CREB.

**Conclusion:**

These findings suggest the involvement of the AdoS in leprosy neuropathogenesis and support the idea that *M. leprae*, by downmodulating the expression and activity of A_2A_R in Schwann cells, decreases A_2A_R downstream signaling, contributing to the maintenance of LD accumulation and intracellular viability of the bacillus.

## 1 Introduction

Leprosy is a chronic infectious disease that can lead to severe peripheral neuropathy. The etiological agent of leprosy is *M. leprae* (Han et al., 2008; Sharma et al., 2013; Romero-Navarrete et al., 2022), although a possible second agent, *M. lepromatosis*, has been identified (Deps and Collin, 2021). *M. leprae* has been detected inside both non-myelinating and myelinated Schwann cells (SCs) in the nerves of leprosy patients (Scollard et al., 2015; Ma et al., 2023). The SCs are the glial cells of the peripheral nervous system (PNS) responsible for the maintenance of the myelin sheath and neuronal homeostasis and the plasticity of the PNS (Gomez-Sanchez et al., 2015). Literature shows that *M. leprae* is intimately related to the demyelinating phenotype associated with peripheral nerve injury, a hallmark symptom of leprosy patients that can be irreversible and results in physical deformities, disability, and stigmatization of patients (Lockwood, 2019). Despite the participation of immune cells in this phenotype (Masaki et al., 2015; Pinheiro et al., 2018), demyelination and axonal injury can occur at the onset of *M. leprae* infection, in the absence of immune cells (Rambukkana et al., 2002).

*M. leprae* actively modulates the SC metabolism to establish a safe intracellular niche for its survival (Ng et al., 2000; Rambukkana et al., 2002; Tapinos and Rambukkana, 2005; Ebenezer and Scollard, 2021). Several studies have shown that *M. leprae* subverts host metabolic pathways and cellular processes to ensure its survival and the establishment of the infection (Medeiros et al., 2016; de Macedo et al., 2020). Among the metabolic changes induced by *M. leprae*, the impact on the host’s lipid homeostasis is noteworthy. *M. leprae* increases the uptake of exogenous cholesterol in infected macrophages and SCs, leading to the accumulation of lipid droplets (LDs) (Mattos et al., 2011a; Mattos et al., 2011b; Mattos et al., 2014) Our group showed that cholesterol biosynthesis is higher in multibacillary leprosy skin biopsies than in paucibacillary lesions, and this phenotype is associated with higher levels of sterol regulatory element-binding protein (SREBP1/2) transcriptional factors—crucial regulators of genes involved in biosynthesis and uptake of cholesterol (Mattos et al., 2014). Furthermore, *in vitro* and *in vivo* studies show that statins and cholesterol synthesis inhibitors decrease intracellular bacterial viability (Mattos et al., 2014; Lobato et al., 2014). Another important regulator of lipid and glucose metabolism is the transcription factor peroxisome proliferator-activated receptor gamma (PPARγ), which is also upregulated both in macrophages and SCs infected by *M. leprae* (Mattos et al., 2012; Díaz Acosta et al., 2018).

The contribution of the purinergic system (PS) as a modulator of the peripheral glia functioning during both development and pathological conditions has been described and has provided targets for the treatment of various pathologies (Fredholm et al., 2011; Burnstock, 2017). The PS consists of ecto-nucleotidases, which regulate the extracellular levels of nucleotides and nucleosides such as adenosine triphosphate (eATP) and adenosine (eADO), the main mediators of this system that act on the purinergic receptors (Mello et al., 2014; Huang et al., 2021). ADO is an intracellular metabolite of purine metabolism; however, the extracellular levels can be increased under conditions such as infection, inflammation, and tissue damage (Antonioli et al., 2013; Morandi et al., 2018). In these circumstances, ATP is leaked out and sequentially metabolized to eADO by two ecto-nucleotidases: CD39 and an ecto-5′-nucleotidase (NT5E/CD73) (Antonioli et al., 2014; Giuliani et al., 2021). eADO can be further metabolized to extracellular inosine (eINO) by ecto-adenosine deaminase (ecto-ADA) (Cristalli et al., 2001). The activation of different biological effects by eADO occurs via the P1 receptors A_1_, A_2A_, A_2B_, and A_3_ (Sheth et al., 2014) coupled to G proteins, stimulatory (A_2A_R/A_2B_R) or inhibitory (A_1_R/A_3_R). A_2A_R stimulates the production of cyclic AMP (cAMP) (Borea et al., 2018; Huang et al., 2021) which, through protein kinase A (PKA) activation, modulates several downstream effectors such as the cAMP-responsive element binding protein (CREB), a transcriptional regulator of several target genes, among them, genes that encode cytokines and lipid metabolism proteins (Li et al., 2015; Giambelluca and Pouliot, 2017; Yoon et al., 2021). A_2A_R has a dual role in being associated with neuroinflammation (Ingwersen et al., 2016) and promyelinating phenotype (Welsh and Kucenas, 2018; De Nuccio et al., 2019). In addition, A_2A_R can modulate lipid metabolism by decreasing foam cell formation and by enhancing the cholesterol efflux via the upregulation of reverse cholesterol transporters (Bingham et al., 2010; Koupenova et al., 2012; Koupenova and Ravid, 2013). The involvement of this system has been extensively explored in the context of infectious diseases (Dubois-Colas et al., 2014; Petit-Jentreau et al., 2015; Basu et al., 2020; Pietrobon et al., 2022), although there are no studies in the context of leprosy.

In the present study, we show that *M. leprae* infection increases the expression and enzymatic activity of NT5E*/*CD73 and expression of ecto-ADA but it downregulates the expression and activity of A_2A_R in SCs. Interestingly, experimental A_2A_R activation reverses the accumulation of LDs induced by *M. leprae* by decreasing the expression of pro-lipogenic genes such as *PPARγ* and *SREBF1* and increasing *ABCA1,* a gene that encodes reverse cholesterol transporter. Additionally, the activation of A_2A_R modulates the production of two inflammatory cytokines, IL-6 and IL-8, and decreases intracellular *M. leprae* viability. These observations provide the first evidence that AdoS components are relevant in the *M. leprae*-SCs interaction. They also increase our understanding of leprosy’s neuropathogenesis and may provide potential new targets for developing complementary therapeutic strategies for disease control.

## 2 Materials and methods

### 2.1 Reagents and antibodies

Oil Red O (ORO/O0625), adenosine (ADO/A9251), adenosine 5′-(α,β-methylene) diphosphate (AMP-CP/M3763), adenosine 5′-monophosphate disodium salt (5′ AMP/01930), 8-[4-[((4-cyanophenyl)carbamoylmethyl)oxy]phenyl]-1,3-di (n-propyl)xanthine hydrate (MRS 1754/M6316), CGS21680 (ab120453), MRS1754 (264622-58-4), ZM 241385, Tris (hidroximetil)-aminometano (Tris-HCL/93363), NP-40 (492016), sodium deoxycholate (SDS/D6750) and [3-(4,5-dimethylthiazol-2-yl)-2,5-diphenyltetrazolium bromide] (MTT/M6494), TritonX-100 (HFH10), bovine serum albumin (BSA/Sigma A2153), trichloroacetic acid (TCA/ThermoFisher), and potassium phosphate (KH2PO4/P9791**)** were purchased from Sigma-Aldrich, USA. It was also purchased: cOmplete™ EDTA-free Protease Inhibitor Cocktail (04693132001) from Roche, United States; anti-ADA (sc-28346) and anti-GAPDH (sc-32233) from Santa-Cruz, USA; LIVE/DEAD™ Bac Light™ Bacterial Viability kit, Bicincichonic acid kit, 4′,6-Diamidino-2-fenilindol,2-(4-Amidinofenil)-6-indolecarbamidina (DAPI/D1306), Prolong Gold Antifade Mountant (36980), RPMI 1640 medium, (2-hydroxyethyl)-1-piperazineethanesulfonic acid (HEPES), L-Glutamine, MemCode™ Reversible Protein Stain Kit (24580), TRIzol^®^, SYBR Green^®^ PCR master mix, and a DNA-free turbo kit from ThermoFisher Scientific, United States; fetal bovine serum (FBS 0010S/Cripion Biotecnology), anti-mouse CD73 (Ab-54217), anti-mouse Alexa Fluor®-488 (ab150117), and anti-rabbit Alexa Fluor®-594 (ab150080) from Abcam, United Kingdom; anti-rabbit A_2A_ receptor (AAR002), anti-rabbit A_2B_ (AAR-004), anti-rabbit A_1_ (AAR-006), and anti-rabbit A_3_ (AAR-004) from Alomone, anti-p-CREB (ab32096); anti-CREB (ab31387) from Abcam, United States; GoScript Master Mix from Promega.

### 2.2 Mycobacteria strain

The live *M. leprae* Thai-53 strain used throughout the study was isolated from the hind footpads of athymic *nu/nu* mice kindly provided by Dr. Patrícia Sammarco Rosa and Ms. Daniele Bertolucci of Instituto Lauro de Souza Lima, Bauru, SP, Brazil. Briefly, about 9 months after inoculation of the bacilli (with a bacillary load of about 10^9^–10^10^/g of tissue), the mice were euthanized, and *M. leprae* was purified from their footpads (Trombone et al., 2014). Viability was checked by microscopy using LIVE/DEAD™ Bac Light™ Bacterial Viability Kit following the manufacturer’s guidelines, and the bacilli were quantified before *in vitro* experiments (Trombone et al., 2014). All procedures were conducted under the *Laboratory Animals Welfare Act*, CONCEA Guidelines, and Policies for Rodent experiments provided by Ministry of Science and Technology of Brazil and the Institutional Animal Care and Use Committee (Approval CEUA-ILSL 00).

### 2.3 Human Schwann cell culture

Human Schwann cells (SCs) from the ST88-14 lineage were used in this study. This SC line was derived from patients with neurofibromatosis type 1 and was generously provided by Dr. Jonathan A. Fletcher from the Department of Pathology at Brigham and Women’s Hospital, Harvard Medical School, Boston, USA. ST88-14 cells were cultured in RPMI 1640 medium supplemented with 10% FBS, 10 mM HEPES, and 1 mM L-Glutamine, pH 7.4. Cultures were maintained at 37 °C with 5% CO_2_ in 150 cm^2^ flasks until they reached approximately 80% confluence.

### 2.4 *Mycobacterium leprae*–Schwann cell infection assays

SCs were suspended in RPMI 1640 culture medium in 2% FBS without antibiotics and seeded at a density of 5 × 10^3^ cell/well on 96-well plates for cell viability assays, 3 × 10^5^ cells/well on 6-well plates for RT-qPCR assays, and at 5 × 10^4^ cells/well on 24-well plates for enzymatic activity assays, fluorescence microscopy experiments, and Western blotting analysis. To investigate the desired outcomes, the cells were stimulated with different compounds 30 min prior to infection with live *M. leprae*. The compounds used were: 5′AMP (100 µM), a substrate of CD73 enzyme; AMP-CP (5 µM), a selective CD73 inhibitor; adenosine (100 µM) and CGS21680 (100 µM), non-selective and selective agonists of the A_2A_R, respectively; ZM132485 (1 µM) and MRS1754 (1 µM), non-selective and selective antagonists of A_2A_R and A_2B_R, respectively. Cells were subsequently infected with *M. leprae* at a multiplicity of infection (MOI) of 5:1 or 50:1 and incubated at 33 °C with 5% CO_2_. The biological responses were assessed at 12, 24, or 48 h post-infection according to the assay to be performed.

### 2.5 Cellular toxicity

To evaluate drug toxicity in SCs in response to compounds used such as 5′AMP (100 µM), AMP-CP (5 µM), CGS21680 (100 µM), ZM132485 (1 µM), and MRS1754 (1 µM), the MTT assay was used. SCs were plated into 96-well plates (10^3^ cells/well). After 48 h, MTT was added, and the formation of formazan was measured (Mosmann, 1983).

### 2.6 Immunofluorescence microscopy

For immunofluorescence analysis, 4 × 10^4^ SCs were plated on glass coverslips in RPMI supplemented with 10% FBS and infected or not with live *M. leprae* (MOI 5:1) for 24 or 48 h. The cells then were fixed with 4% paraformaldehyde (PFA). They were subsequently incubated with a permeabilization solution composed of phosphate buffer saline (PBS) 1X, 10% FBS, and 0.001% Triton X-100 and were incubated with blocking solution containing 1% BSA diluted in PBS 1X with 0,001% Triton. The expression of CD73, ADA, and A_2A_R was evaluated using mouse monoclonal anti-CD73, rabbit polyclonal anti-ADA, and rabbit polyclonal anti-A_2A_R, all at a dilution of 1:250 and incubated overnight at 4 °C. The slides were washed thrice with PBS and incubated with Alexa Fluor^®^ conjugated secondary antibodies (anti-mouse-Alexa Fluor^®^-488 or anti-rabbit-Alexa Fluor^®^-594) for 1 h at a dilution of 1:1000. Nuclear staining was done using DAPI labeling (1:100). Coverslips were mounted using prolong gold antifade mounting. Images were captured with a Zeiss Axio Observer inverted microscope using the Colibri illuminating system with Plan-Neofluar ×40 objectives (Zeiss, Oberkochen, Germany) equipped with AxioVision Rel. 4.8 software (Zeiss, Oberkochen, Germany). Quantification was performed using ImageJ software (Schneider et al., 2012) measuring the fluorescence area per cell number in each image.

### 2.7 Lipid droplet staining and quantification

To investigate LDs accumulation, SCs were plated in RPMI supplemented with 2% FBS. The cells were treated with ADO, CGS21680, ZM132485, or MRS1754 as described for the SC infection assays and infected or not with *M. leprae* (MOI 5:1) for 48 h. Cells were stained with Oil Red O (ORO) (Díaz Acosta et al., 2018). The coverslips were washed with distilled water, and the cells’ nuclei were labeled with DAPI. Coverslips were mounted using prolong gold antifade mounting. The images were observed under an Axio Observer Z1 fluorescence microscope equipped with the AxioVision Rel. 4.8 software. The images were made with a 40 × objective lens and at least ten fields per slide; a total of 200 cells per condition were photographed. The quantification of LDs was performed using ImageJ software, with the Macro Umbral plugin as previously described Díaz Acosta et al. (2018). Macro Umbral calculated the lipid droplet area which was divided by the number of cells present in the analyzed field to determine the LD quantification (LD area/cell). Graphs demonstrate the mean value of LD/cell per condition.

### 2.8 Ecto-5′Nucleotidase activity assay

CD73 activity was measured by colorimetric method with malachite green by the rate of released inorganic phosphate (Pi) (Zanin et al., 2012; Allard et al., 2019). Specifically, 5 × 10^4^ SCs were plated on 24-well plates and infected or not with *M. leprae* (MOI 5:1 or 50:1) for 24 h at 33 °C with 5% CO_2_, in the presence or not of the CD73 inhibitor AMPCP (5 µM). The supernatant was discarded, and the wells were thrice washed with reaction medium (phosphate free). The reaction was started by the addition of 500 µL of reaction medium containing 2 mM of CaCl_2_, 120 mM NaCl, 5mM KCl, 10 mM glucose, 20 mM Hepes, pH 7.4 pH, and 5 mM 5′AMP, and they were incubated for 1 h at 25 °C. The cell incubation medium (500 μL) was removed and centrifuged at 5,000 × g for 5 min. The supernatant was transferred to a tube containing 500 µL of 5% (w/v) trichloroacetic acid (TCA) on ice. Inorganic phosphate (Pi) production was measured by absorbance of the phosphomolybdate-malachite at 630 nm. The standard curve of Pi was made with a solution of KH_2_PO_4_, and CD73 activity was calculated by subtracting the nonspecific 5′-AMP hydrolysis measured in the absence of cells. The concentration of Pi released in the reaction was determined by comparison with a standard curve. All samples were taken in biological triplicates, and the CD73 activity was expressed as pmol Pi × min^-1^× 10^−4^ cells (Zanin et al., 2012; Allard et al., 2019).

### 2.9 Western blot

To evaluate protein expression, SCs were plated as described for the SC infection assays, treated or not with CGS21680 and infected or not with *M. leprae* (MOI 50:1) for 48 h. The cells were lysed using a RIPA buffer (50 mM TRIS pH 7.5, 1% NP-40, 0.25% sodium deoxycholate, 0.1% SDS) supplemented with protease inhibitors. Protein concentrations were determined using the BCA Kit. For SDS-PAGE electrophoresis, 20 µg of protein was loaded onto a 12% polyacrylamide gel, and the efficiency of the transfer was confirmed by staining the nitrocellulose membrane with MemCode™ Reversible Protein Stain Kit. The primary antibodies used were anti-A_2A_R, (dilution 1:500) and anti-p-CREB (1:500 dilution). Anti-GAPDH (dilution 1:1000) was used as the loading control. Primary antibodies were incubated overnight at 4 °C. After three sequential washes with TBS-Tween and TBS 5 min each, the HRP-conjugated secondary antibodies were incubated 1 h. Chemiluminescence detection was performed using the iBright™ CL1500 Imaging System (A44114), and the relative protein levels were determined using ImageJ software.

### 2.10 Real-time quantitative reverse transcription PCR (RT-qPCR)

SCs were infected with *M. leprae* (MOI 5:1) overnight at 33 °C in the presence or absence of CGS21680 (100 µM); the mRNA was isolated using TRIzol^®^ according to the manufacturer’s instructions. Briefly, the nucleic acid content was separated, followed by precipitation of RNA and subsequent production of cDNA. To assess the integrity of the RNA samples, a 1.2% agarose gel electrophoresis technique was employed. Samples showing two corresponding distinct bands without any signs of degradation were considered suited for subsequent analysis. The RT-qPCR was performed to analyze differential gene expression. The sequences of primers are available in Supplementary Table S1. Reverse transcription was performed using GoScript Master Mix. The RT-qPCR reactions were performed in duplicates on a ViiA7 Real-Time PCR System (Applied Biosystems, USA) using Power SYBR Green Master Mix. For this, the cDNA samples were diluted to a concentration of 5 ng/μL, and PCR mix was prepared for application in a 384-well plate. The relative gene expression analysis was performed using adjusted efficiency. Briefly, raw data were exported from ViiA7 software and imported into LinRegPCR v.2022 (Ramakers et al., 2003). Data analysis was carried out using the comparative method *ΔΔ*CT (Livak and Schmittgen, 2001) after normalization with the endogenous housekeeping gene *RPL13.* Normalized expression values were presented as Fold Change (FC). To take into account differences between biological replicates of NI samples, the mean of all biological replicates was determined along with the ratio between each biological replicate and this mean. Therefore, the NI condition is represented as a mean ± SD.

### 2.11 Bacterial viability

*M. leprae* viability was investigated by RT-qPCR as described by Martinez et al. (2009). Briefly, SCs pre-treated or not with CGS21680 (100 µM) were infected with *M. leprae* for 48 h. RNA was extracted as described in Section 2.10, and DNA was extracted as described in Ramakers et al. (2003). DNA was removed from the RNA samples using DNA-free turbo kit, and the RNA reverse transcription was performed using GoScript Master Mix according to the manufacturer’s instructions. The levels of 16S rRNA were calculated against 16S DNA. LinRegPCR software was used to calculate the PCR efficiency for each biological experiment (Martinez et al., 2009), and the normalization was made considering the efficiency corrections. The primers for the rRNA 16S were used for both cDNA and DNA: sense 5′-GAA ACT GCG AAT GGC TCA TTA AAT CA-3^’^ and antisense 5^’^- CCC GTC GGC ATG TAT TAG CTC T-3^’^. Both cDNA and DNA were measured by TaqMan real-time PCR assay (TaqMan probe 16S 5^’^-TGG TTC CTT TGG TCG CTC GCT CC-3^’^). *M. leprae* viability was determined by the comparative Ct method described by Martinez et al. (2009), and viability was calculated as described by Díaz Acosta et al. (2018). Cycling and plate reading were conducted using the Viia7 machine with Real-Time PCR System software.

### 2.12 Enzyme-linked immunosorbent assay (ELISA)

SCs cultures were infected by *M. leprae* (MOI 5:1), and the supernatants were collected after 48 h infection for ELISA analysis. The levels of interleukin 6 and 8 (IL-6 and IL-8) were assessed using ELISA with respective Duo-Set human ELISA kits (R&D Systems) following the manufacturer’s guidelines. Optical densities were measured by an Absorbance microplate reader (EON BioTek Instruments), and the data were analyzed using SoftMax R Pro Software version 5.3 (ThermoFisher).

### 2.13 Interactome analysis

The protein interactome network was generated used STRING (https://string-db.og) version 12.0. The search was done by multiple proteins using *Homo sapiens* databank. A full STRING network was selected as the network type, with a required score of medium confidence (0.400), and medium false discovery ratio (FDR) stringency corresponding to 5%. Markov clustering (MCL) was applied—a clustering algorithm for graphs that assigns proteins into families based on sequence similarity information with 3.0 of inflation parameters.

### 2.14 Statistical analysis

Data statistical analysis was conducted using Graph Prism software version 9.0.0, and statistical significance was determined by a two-tailed unpaired *t*-test for single comparisons or one-way ANOVA tests for multiple comparisons, followed by Bonferroni corrections. The statistical differences between the groups were inferred to be significant when *p < 0.05, **p < 0.01, and ***p < 0.001.

## 3 Results

### 3.1 *Mycobacterium leprae* infection upregulates both CD73 and ADA enzymes in Schwann cells

**FIGURE 1:**
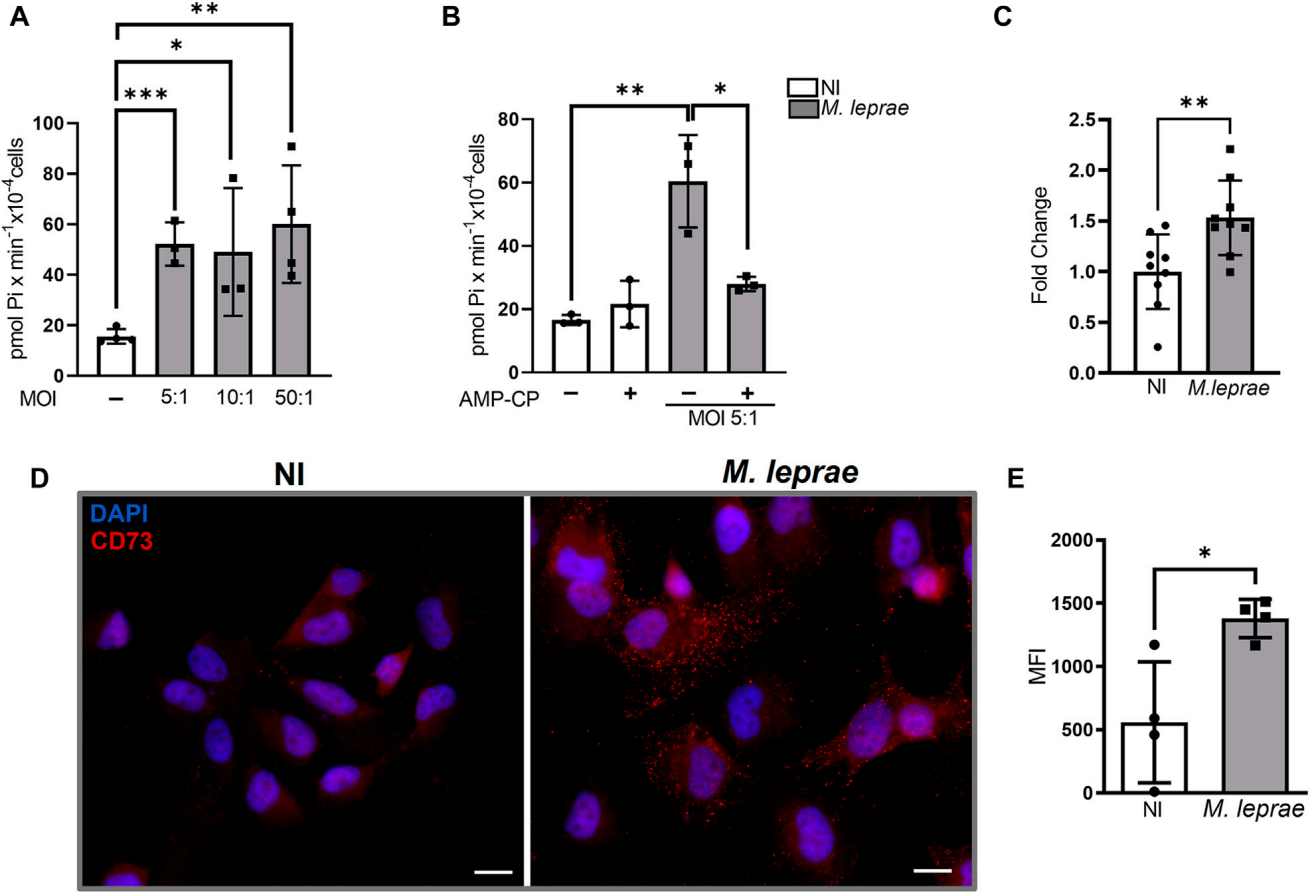
Ecto-5′ nucleotidase-CD73 is upregulated in Schwann cells infected with *Mycobacterium leprae.* **(A,B)** 5 × 10^4^ SCs were infected with *M. leprae* for 24 h. 5′AMP (5 mM) was added and incubated for 1 h at 25°C. Ecto-5′nucleotidase activity was measured through Pi release using a malachite green colorimetric assay and expressed as pmol Pi x min^-1^x 10^−4^ cells. **(A)** *M. leprae* increased the 5′AMP hydrolysis at different MOIs (5:1, 10:1, and 50:1). **(B)** AMP-CP (5 µM), a specific CD73 inhibitor, reversed the effect of *M. leprae* (MOI 5:1) on the 5′AMP hydrolysis in infected SCs. **(C)** *M. leprae*-infected SCs, 16 h post-infection, increased expression of *NT5E* mRNA, normalized by *RPL13*, measured by RT-qPCR. **(D,E)** Immunofluorescence assay showing increased expression of CD73 enzyme in SCs 24 h post-infection. SCs infected with *M. leprae* (MOI 5:1) were treated with anti-CD73 antibodies (red). DAPI used for nuclear staining (blue). Scale bar: 20 µm. **(E)** Mean fluorescence intensity (MFI) quantified using ImageJ software. NI = not infected. All data mean ± SD. Statistical significance determined using parametric Student’s t-test: *p < 0.05, **p < 0.01, and ***p < 0.001.

Extracellular ADO (eADO) signaling is regulated through different mechanisms, including CD73 and ecto-ADA, that convert extracellular 5′AMP to eADO and eINO, respectively (Huang et al., 2021). To investigate the impact of *M. leprae* infection on AdoS, 5′ AMP hydrolysis, the levels of Pi were measured via colorimetric assay (Zanin et al., 2012; Allard et al., 2019). Initially three multiplicity-of-infection (MOI) ratios (5:1, 10:1, and 50:1) were assayed. The results revealed a significant increase in extracellular 5′AMP hydrolysis in infected SCs (Figure 1A) regardless of MOI. *M. leprae-*infected SCs were 52.21 ± 8.553 pmol Pi × min^-1^×10^−4^cells (MOI 5:1/*p* = 0.0004), 49.09 ± 25.27 pmol Pi × min^-1^×10^−4^cells (MOI 10:1/*p* = 0.0422), and 60.02 ± 23.27 pmol Pi × min^-1^×10^−4^cells (MOI 50:1/*p* = 0.0004) —about three to four times more than the not infected (NI) SCs (Figure 1A). Since no statistical difference was observed in the extracellular hydrolysis of 5′AMP between the different MOIs, the ratio 5:1 was adopted in the remaining experiments. To confirm that hydrolysis of extracellular 5′ AMP resulted from CD73 activity, a non-hydrolysable 5′AMP analogue, adenosine-5'-(α, β-methylene) diphosphate (AMP-CP) —a selective CD73 inhibitor—was used. When applied to the infected cells, it reduced Pi production by 46.8% (*p* = 0.019) (Figure 1B). AMP-CP’s cytotoxicity was evaluated by MTT assay and did not affect the viability of SCs (data not shown). In addition to CD73 activity, we also analyzed the impact of *M. leprae* infection on *NT5E* gene expression which encodes CD73, and we observed a 1.18 log_2_ fold change compared to the NI condition (FC = 1.000 ± 0.3681 and *M. leprae* FC = 1.532 ± 0.3677) (*p* = 0.0074) (Figure 1C). Immunofluorescence showed diffuse immunostaining of CD73 on *M. leprae-*infected cells (MIF = 821.3 ± 250.9) with a 1.470-fold change compared to the NI condition (MIF = 555.8 ± 478.7) (*p* = 0.0170) (Figures 1D, E).

**FIGURE 2:**
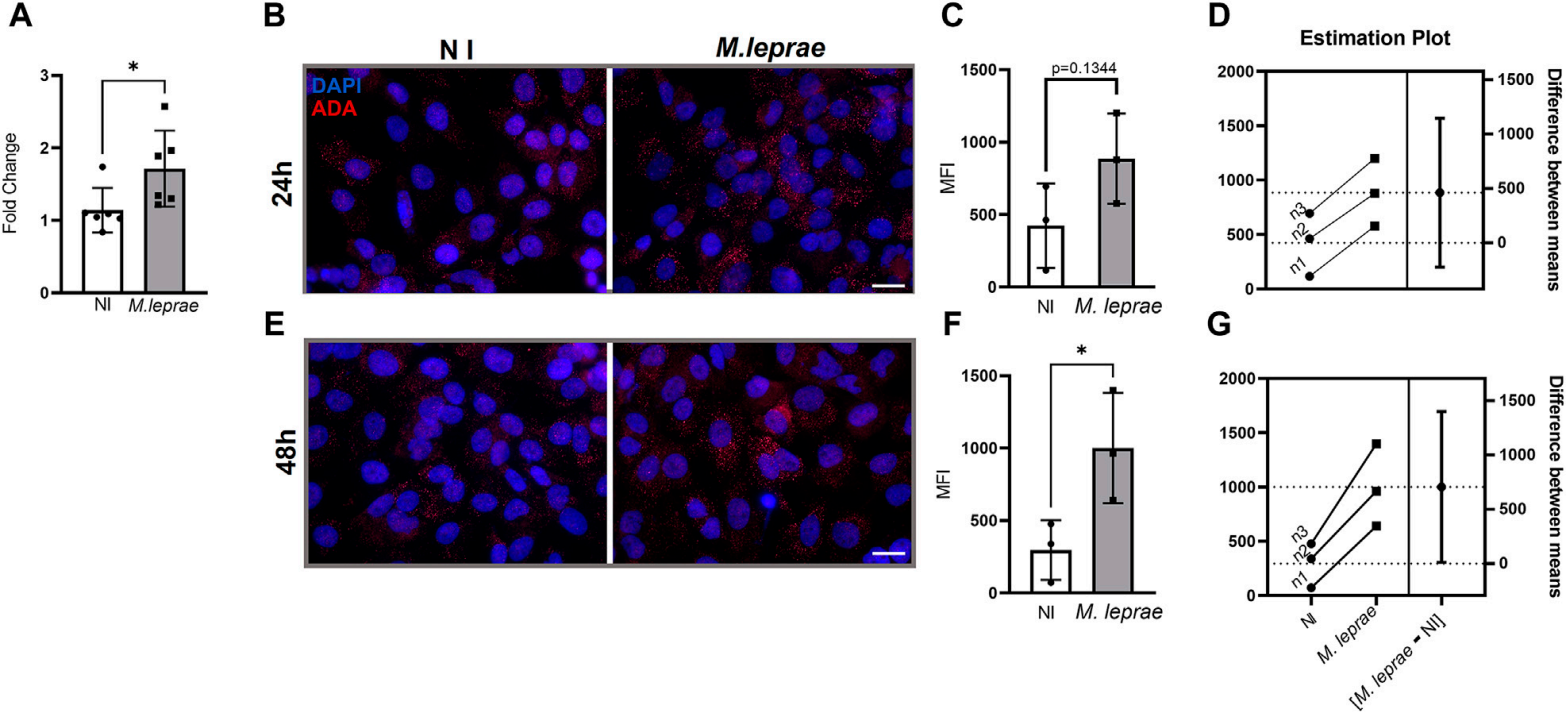
*Mycobacterium leprae* induces expression of the enzyme adenosine deaminase (ADA) in Schwann cells. **(A–C)** SCs were infected with *M. leprae* at a MOI of 5:1. **(A)** *M. leprae* increased *ADA* mRNA expression levels in SCs after 16 h of infection. For this, 3 × 10^5^ SCs were infected, and gene expression was assessed via RT-qPCR. The *RPL13* gene was used as the endogenous control gene. **(B–G)** Immunofluorescence analysis and quantification of ADA expression. 4 × 10^4^ SCs infected with *M. leprae* for 24 h **(B–D)** or 48 h **(E–G)** were stained with antibody against ADA (red) and DAPI as nuclear stain (blue). Scale bar: 20 µm. **(C,F)** Mean fluorescence intensity (MFI) was quantified using ImageJ software. **(D,G)** Estimation plot showing that *M. leprae* induced ADA expression in SCs 24 h and 48 h post-infection in the three biological replicates; however, in **(C)** the statistical difference was not reached. **(E)** *M. leprae* significantly increased the expression of the ADA enzyme in SCs 48 h post-infection. NI = not infected. All data mean ± SD. Statistical significance determined using parametric Student’s t-test: *p < 0.05.

We also analyzed the impact of *M. leprae* infection on *ADA* gene expression. Figure 2A shows that infected SCs exhibited an increase of 20.9% in *ADA* gene expression (FC 1.714 ± 0.5254) in comparison to NI SCs (FC = 1.140 ± 0.3081) (*p* = 0.0436). ADA protein was analyzed by immunofluorescence 24 and 48 h post-infection (Figures 2B–F). No statistical difference at 24 h infection was found due to the variability between biological replicates. However, the estimation plot (Figure 2D) revealed that all M*. leprae*-infected biological replicates exhibited higher ADA levels than NI SCs (Figures 2B–D). At 48 h post cellular infection, ADA levels increased with a fold change of 3.37 (MIF = 1001 ± 381.1) compared to the NI condition (MIF = 296.3 ± 206.1) (*p* = 0.0480) (Figures 2E–G). Taken together, these data indicate that *M. leprae* infection upregulates both CD73 and ADA, responsible for metabolizing extracellular eAMP to eINO, potentially contributing to the reduction of eADO levels in infected SCs (Supplementary Tables S2–S4 compiles the data presented in this section).

### 3.2 *Mycobacterium leprae* infection downregulates the expression of A_2A_R receptor in Schwann cells

**FIGURE 3:**
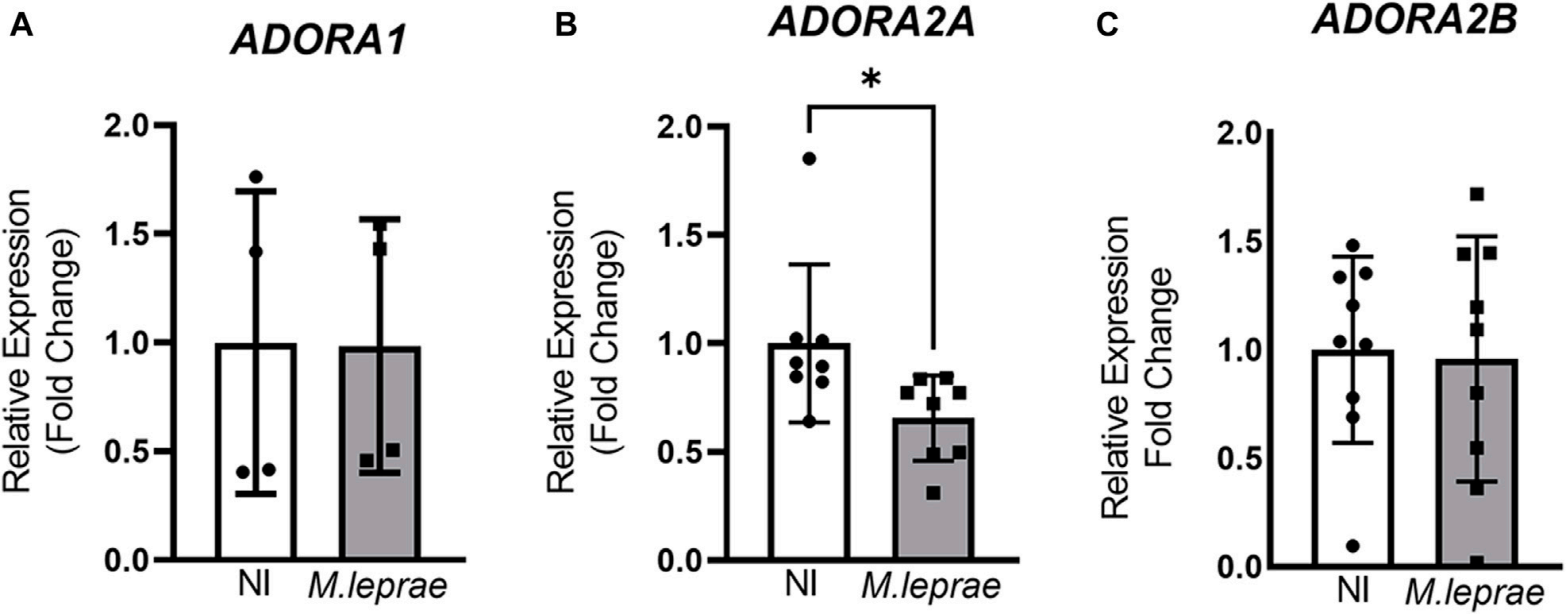
Downregulation of *ADORA2A* gene expression in *M. leprae*-infected Schwann cells; 3 × 10^5^ SCs were infected with *M. leprae* at MOI 5:1, and the expression of the genes **(A)** *ADORA1*, **(B)** *ADORA2A*, and **(C)** *ADORA2B* was assessed via RT-qPCR after 16 h of infection. *RPL13* was used as endogenous control gene. All data are mean ± SD. Statistical significance determined using parametric Student’s t-test: *p < 0.05.

**FIGURE 4:**
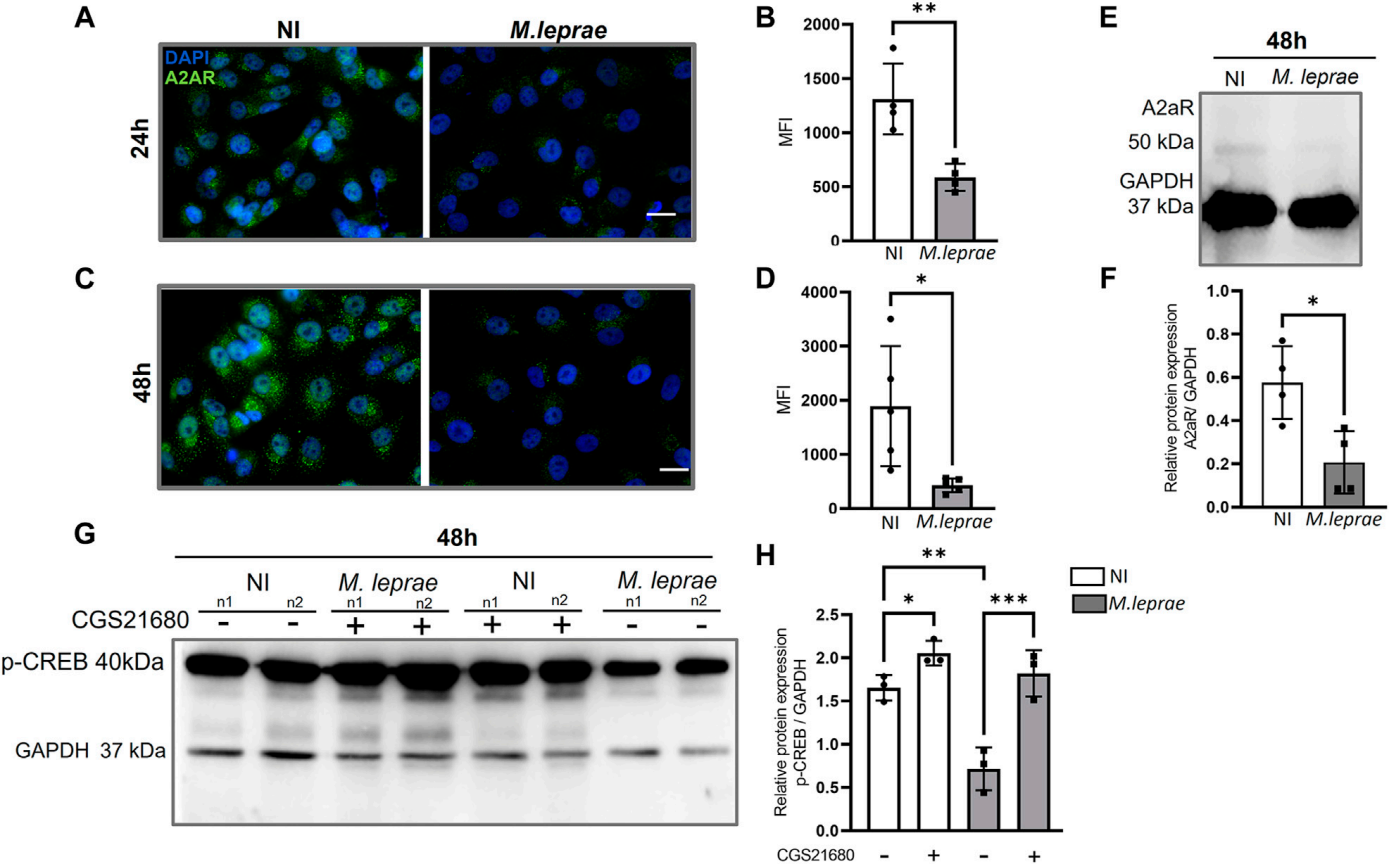
A_2A_ receptor downregulated in Mycobacterium leprae-infected Schwann cells. **(A,C)** 4 × 10^4^ SCs infected with M. leprae (MOI 5:1) for 24 and 48 h respectively. Significant reduction in the expression of the A_2A_ was observed. Protein expression of A_2A_R (green) visualized using immunofluorescence. DAPI nuclear staining in blue. Scale bar: 20 μm. **(B,D)** Mean fluorescence intensity (MFI) quantified using ImageJ software. **(E)** 1.5 × 10^5^ SCs infected with *M. leprae* (MOI 5:1) for 48 h, A_2A_R protein expression analyzed by Western blot. **(F)** Relative densitometry values for A_2A_R expression. **(G)** SCs infected with *M. leprae* (MOI 5:1) for 48 h, p-CREB protein expression analyzed by Western blot and the A_2A_R specific agonist, CGS21680 (100 μM) was used to evaluate the impact of A_2A_R activation on p-CREB expression. **(H)** Densitometry values of p-CREB protein expression normalized using endogenous GAPDH. NI = not infected. All data mean ± SD. Statistical significance was determined using parametric Student’s *t*-test: *p < 0.05 and **p < 0.01.

Extracellular ADO activates a family of P1 receptors, such as A_1_R, A_2A_R, A_2B_R, and A_3_R, which have different affinity to eADO (Patritti-Cram et al., 2021). To understand the role of eADO during *M. leprae* infection, we compared the expression profile of these receptors in infected *versus* NI SCs. The experimental data showed that the ST88-14 SC lineage expresses the genes *ADORA1*, *ADORA2A*, and *ADORA2B*. However, there was no significant modulation of the genes *ADORA1* and *ADORA2B* with *M. leprae* infection (Figures 3A, C)*.* In contrast, *ADORA2A* gene expression was downregulated (Figure 3B). The difference between the means of NI versus *M. leprae*-infected SC was −0.3448 ± 0.1464, which represents a reduction of 34.5% (*p* = 0.0336) in *ADORA2A* gene expression in *M. leprae*-infected SCs (Figure 3C) (Supplementary Table S3). The gene *ADORA3* was not detected in this cell line; to confirm this data, we used two primer pairs indicated in Supplementary Table S1. To confirm that *M. leprae* infection reduces A_2A_R levels, immunofluorescence was performed 24 and 48 h post-infection. Consistent with the PCR assay, infection decreased cellular A_2A_R by 55.19% (*p* = 0.0056) and 77.32% (*p* = 0.0098) at 24 and 48 h, respectively (Figures 4A–D). Western blotting analysis confirmed the downregulation by 63.98% of A_2A_R expression 48 h post-infection in SCs, corroborating the PCR and immunofluorescence results (Figures 4E, F).

When activated A_2A_R triggers cAMP-PKA-CREB signaling, it results in the phosphorylation of CREB (p-CREB). To investigate whether A_2A_R could be active despite its downregulation by *M. leprae* infection, we used a specific A_2A_R agonist, CGS21680, to elevate the level of p-CREB. Figures 4G and H show the results of two biological replicates 48 h post-infection; the expression of p-CREB decreased by 56.77% compared to NI (*p* = 0.048) (Supplementary Table S5). Nevertheless, when it was preincubated with CGS21680, that effect was reversed to the level of the NI condition.

### 3.3 Adenosine and the specific A_2A_R agonist, CGS21680, reverse lipid droplet accumulation induced by *Mycobacterium leprae* infection in Schwann cells

**FIGURE 5:**
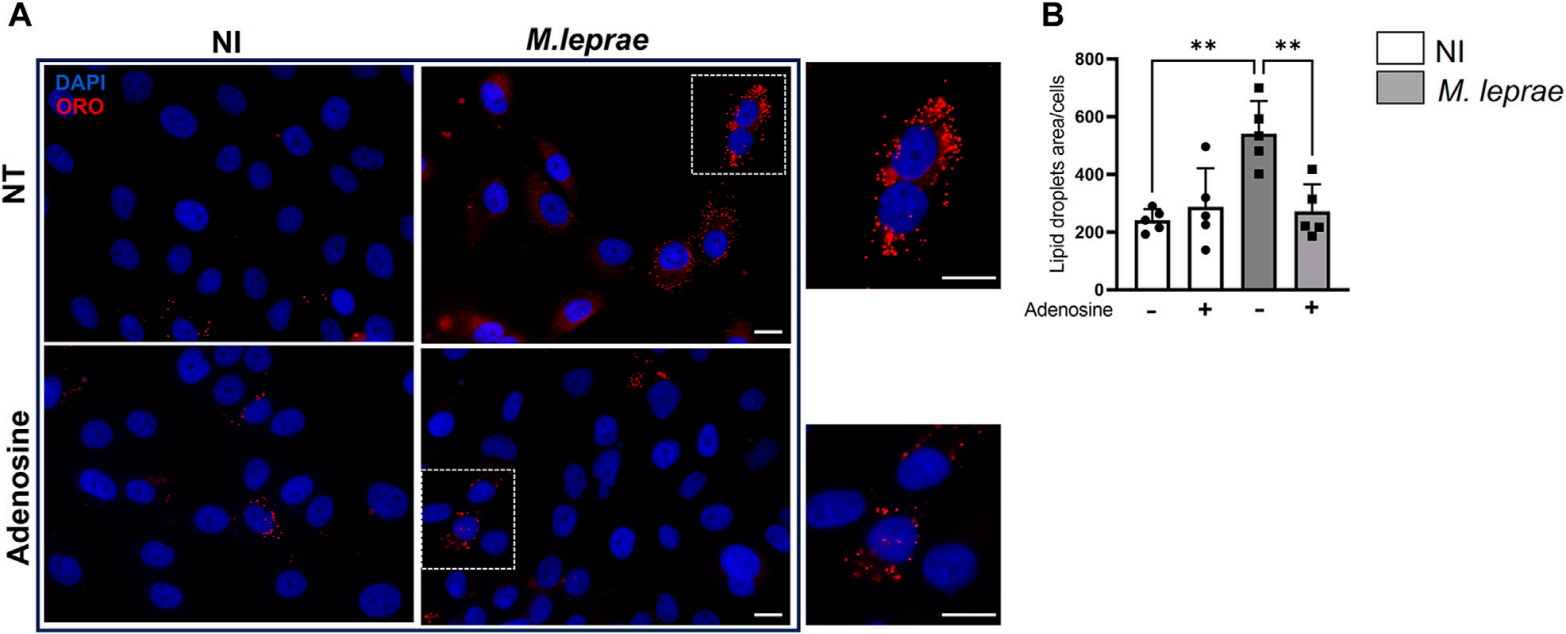
Adenosine decreases *Mycobacterium leprae-*induced lipid droplets accumulation in Schwann cells. 4 × 10^4^ SCs were infected with *M. leprae* (MOI 5:1) for 48 h and LDs were visualized by fluorescence microscopy using Oil Red O (ORO) staining. The quantification of ORO-stained LDs was plotted as measurement of LD area/cell. A total of 200 cells for each condition were analyzed. **(A)** Pretreatment with 100 µM adenosine reduced LD formation induced by *M. leprae*. Scale bar 20 μm (white line). **(B)** LDs quantification by ImageJ software. NT = not treated. Data are shown as mean ± SD of at least three different experiments performed in triplicate. Statistics used the not infected (NI) condition as reference. Data evaluated via parametric Student’s t-test, *p < 0.05, *p < 0.01.

**FIGURE 6:**
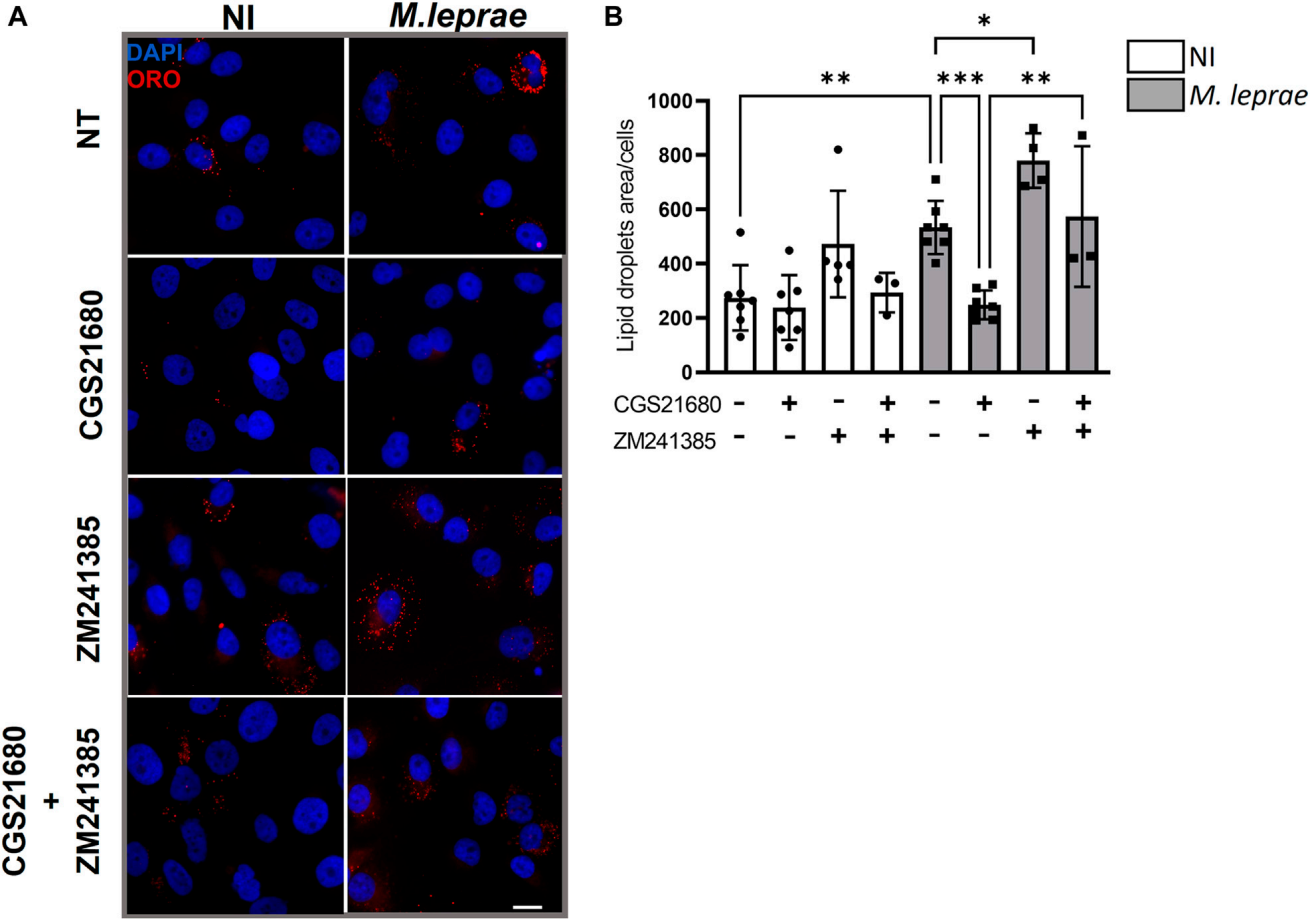
A_2A_ receptor negatively modulates lipid droplet formation in *Mycobacterium leprae-*infected Schwann cells. 4 × 10^4^ SCs were infected with *M. leprae* (MOI 5:1) for 48 h, and LDs were visualized by fluorescence microscopy using ORO staining. The quantification of ORO-stained LDs was plotted as measurement of LD area/cell for a total of 200 cells for each condition. **(A)** Pretreatment with CGS21680 (100 µM) reduced LD formation induced by *M. leprae*. The use of the specific A_2A_R antagonist ZM241385 (1 µM), *per se*, increased the formation of LDs and reversed the effect of CGS21680 in inhibiting *M. leprae-*induced LD. Scale bar 20 μm (white line). **(B)** LDs quantification by ImageJ software. NI = not infected. NT = not treated. Data shown as mean ± SD of at least three different experiments performed in triplicate. Statistical differences evaluated via one-way ANOVA with Bonferroni’s multiple comparisons test, *p < 0.05, *p < 0.01.

A_2A_R can influence cellular cholesterol homeostasis, decreasing LD formation and exhibiting an antiatherosclerosis role (Reiss et al., 2004; Bingham et al., 2010). We thus investigated the possibility that decreased A_2A_R expression induced by *M. leprae* infection was related to the foamy phenotype observed in infected cells. In this context, the impact of A_2A_R on the capacity of *M. leprae* to induce LD formation in SCs was measured. A_2A_R was activated with eADO, and LD formation was measured 48 h post-infection. As expected, *M. leprae* increased LD accumulation with a 3.96-fold change (*p* = 0.0153) *versus* NI SCs. However, when the SCs were treated with eADO, *M. leprae*-induced LD accumulation was significantly inhibited by 73.9% (*p* = 0.0257) (Figures 5A, B). To certify that eADO was activating A_2A_R, a synthetic A_2A_R agonist, CGS21680, was used instead, and this inhibited *M. leprae-*induced LD accumulation at a similar level as eADO (61.40%, *p* = 0.0428). In contrast, a synthetic A_2A_R antagonist, ZM241385, increased the levels of LDs induced by *M. leprae* infection by 78.62% (*p* = 0.0044) compared with *M. leprae* infected and untreated SC cultures. Furthermore, ZM241385 reversed the inhibitory effect of CGS21680 in *M. leprae*-infected cells, (Figures 6A, B). Considering that the EC50 of the compound CGS21680, determined in different studies, is in the nM range (Kull et al., 2000; Gao et al., 2020), we also evaluated the effect of CGS21680 at 50 nM on LDs accumulation induced by *M. leprae*. The result showed that 50 nM CGS21680 continues to inhibit the formation of LDs induced by *M. leprae* in infected SC and that ZM241385 also reversed this phenotype (Supplementary Figures S1A, B). To rule out a possible interference caused by a high concentration of CGS21680 on the A_2B_ receptor, we analyzed the effects of a specific A_2B_R antagonist, MRS1754, on LD formation induced by *M. leprae*. However, we firstly checked the expression of A_2B_R in *M. leprae*-infected SCs and confirmed that infection does not change A_2B_R expression (Supplementary Figures S2A, B). Nonetheless, despite A_2B_R not having its expression modulated by infection, RT-qPCR and immunofluorescence analysis revealed that this receptor is much more abundant in SCs infected by *M. leprae* than A_2A_R, (Figures 3B, C, 4C, D; Supplementary Figures S3A, B). Despite its abundance, we found that MRS1754, unlike ZM241385, did not increase LD formation and was unable to reverse the inhibitory effect of CGS21680 on LD accumulation in *M. leprae*-infected SCs (Supplementary Figures S2A, B). Overall, this analysis provides valuable insights into the participation of A_2A_R as a negative modulator of LD formation in *M. leprae*-infected SCs (Supplementary Table S6 compiles the data presented in this section).

### 3.4 A_2A_R activation exhibits an anti-lipogenic function in *M. leprae*-infected Schwann cells

Our research investigated the crucial role of the A_2A_R receptor in the molecular mechanisms of *M. leprae* infection*.* This receptor, when activated, reverses the formation of LDs induced by *M. leprae* in infected SCs. To explore this, we focused on lipid metabolism genes upregulated by *M. leprae* infection and genes involved in cholesterol efflux upregulated by A_2A_R. Before solidifying our hypothesis, we constructed an interaction network using STRING to assess the interconnection between these genes. We included the cytokines IL-6 and IL-8, modulated by *M. leprae* infection and also involved in lipid homeostasis (Supplementary Figures S4A–C).

The A_2A_R receptor, as we anticipated, interacts with CD73 and ADA enzymes, both of which also interact with IL-6. This cytokine, in turn, interconnects with several targets of lipid metabolism, illustrating the intricate web of molecular interactions. We also highlight the direct connection between A_2A_R and CREB1, which interacts with transcription factors that modulate lipid metabolism, PPARγ, SREBF1, and SREBF2. Furthermore, CREB1 interacts with the cytokines IL-6 and IL-8, which interact with IL-6, PPARγ, and CD36, a gene regulated by PPARγ (Supplementary Figure S4A). Our network’s green and red nodes represent clustered proteins, each with their cellular processes, as shown in color and indicated in Supplementary Figures S4B, C. The green nodes highlight the processes involved in the negative regulation of the inflammatory response and the metabolic process of adenosine, further emphasizing the complexity of the molecular mechanisms at play. The cellular processes selected to red nodes accentuate the regulation of lipid localization and cholesterol storage, lipid metabolic processes, and the cell-surface receptor signaling pathway. Additional information is compiled in Supplementary Table S7.

This interaction network analysis has shed light on potential molecular mechanisms up- or downregulated by A_2A_R that could contribute to decreasing LD accumulation. Importantly, our hypothesis was not just a theoretical construct but was confirmed by rigorous RT-qPCR analysis, adding a layer of confidence to our findings.

**FIGURE 7:**
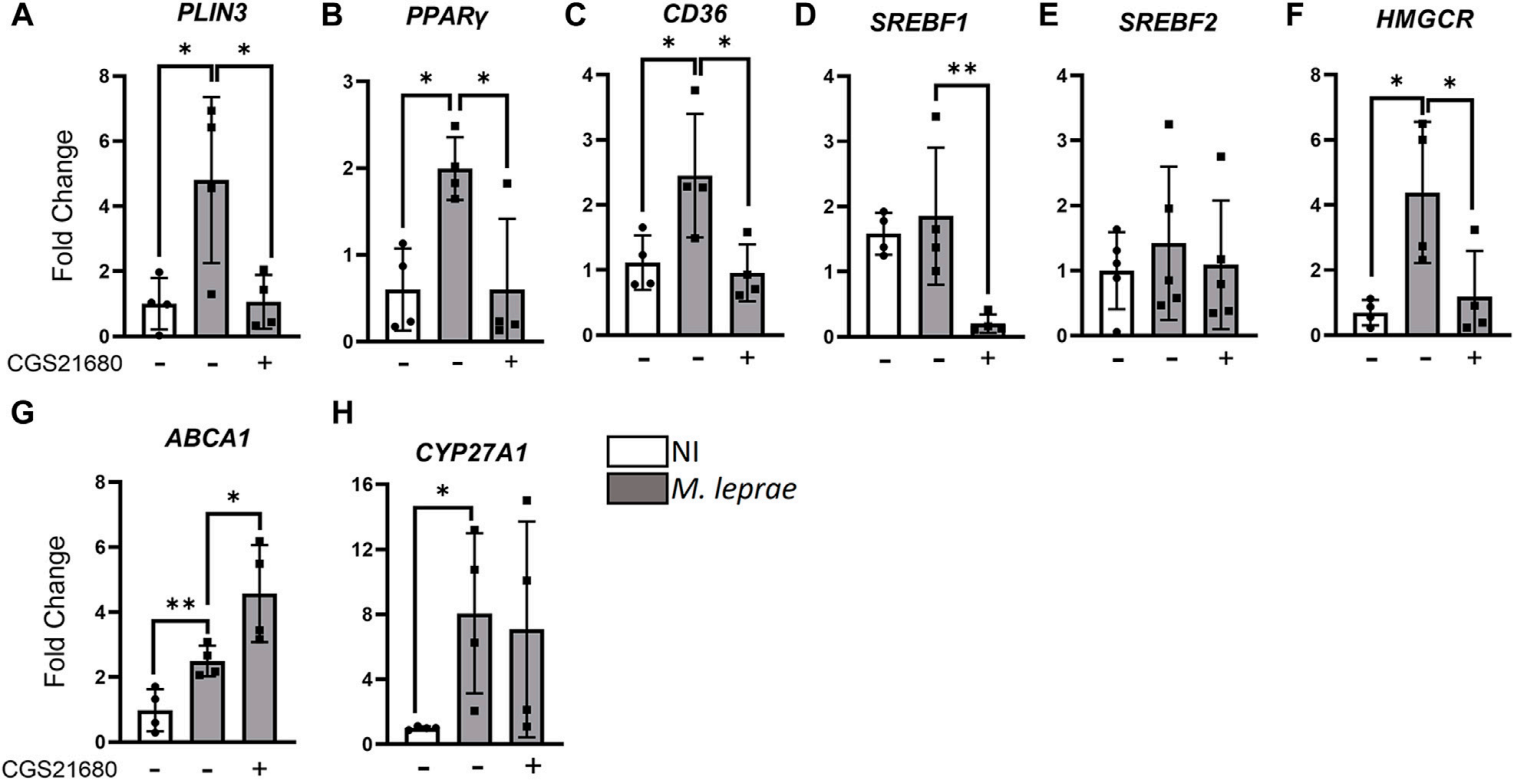
Activation of A_2A_R downregulates expression of pro-lipogenic genes and upregulates genes involved in the cholesterol efflux in *Mycobacterium leprae-*infected Schwann cells. 3 × 10^5^ SCs were infected with *M. leprae* (MOI 5:1) and pre-treated or not with CGS21680 (100 µM). Gene expression was assessed via RT-qPCR after 48 h of infection. The *RPL13* gene was used as the endogenous control. NI = not infected. Data shown as mean ± SD of at least three different experiments performed in triplicate. Statistical significance determined using parametric Student’s t-test: *p < 0.05. Differences assessed by comparing NI *vs M. leprae* and *M. leprae vs M. leprae* + CGS21680 groups. Target genes: **(A)** *PLIN3*; **(B)** *PPARy*; **(C)** *CD36*; **(D)** *SREBPF1*; **(E)** *SREPBF2*; **(F)** *HMGCR*; **(G)** *ABCA1*; **(H)** *CYP27A1*.

Our data showed that *M. leprae* infection upregulated the expression of pro-lipogenic genes, confirming previous results (Mattos et al., 2011a; Mattos et al., 2011b; Mattos et al., 2012; Mattos et al., 2014), while the activation of A_2A_R with CGS21680 reversed this effect (Figure 7). The genes selected for analysis were *PLIN3*, an LD marker; *PPARγ* (peroxisome proliferator-activated receptor gamma), a transcription factor that regulates both adipogenic and lipogenic genes; *CD36*, a cholesterol scavenger receptor, also upregulated by *M. leprae* infection (Figures 7A–C). Moreover, *SREBF1*, which encodes the sterol regulatory-element binding proteins 1, a transcription factor that regulates genes of *de novo* fatty acid synthesis, was also downregulated by CGS21680 (Figure 7D). We did not observe any modulation on the *SREBF2* (Figure 7E); however, *HMGCR*, which encodes 3-hydroxy-3-methylglutaryl CoA reductase, a rate-limiting step in the cholesterol biosynthesis pathway, was also downregulated by CGS21680 (Figure 7F). As well as pro-lipogenic genes, we analyzed the *ABCA1* gene, which is regulated by LXR (liver X receptor) and encodes the ATP-binding cassette protein involved in the elimination of excess cholesterol from the cells. The *CYP27A1* gene, which encodes a sterol 27-hydroxylase that catalyzes the formation of 27-hydroxycholesterol—an oxysterol activator of LXR—was also investigated. The results presented in Figures 7G and H show that *M. leprae* increased the expression of *ABCA1* and *CYP27A1* in infected SCs and that CGS21680 induced a significant increase in the expression of the *ABCA1* gene. Regarding *CYP27A1,* we did not observe any modification when comparing *M. leprae* to *M. leprae +* CGS21680. Supplementary Table S9 summarizes the statistical data and the fold change effects of CGS21680. These findings propose a potential molecular mechanism by which the activation of A_2A_R can reverse the LD accumulation induced by *M. leprae* infection.

### 3.5 Activation of A_2A_R by CGS21680 reverses the production of IL-6 and IL-8 cytokines in *Mycobacterium leprae*-infected Schwann cells and decreases *M. leprae* intracellular viability

**FIGURE 8:**
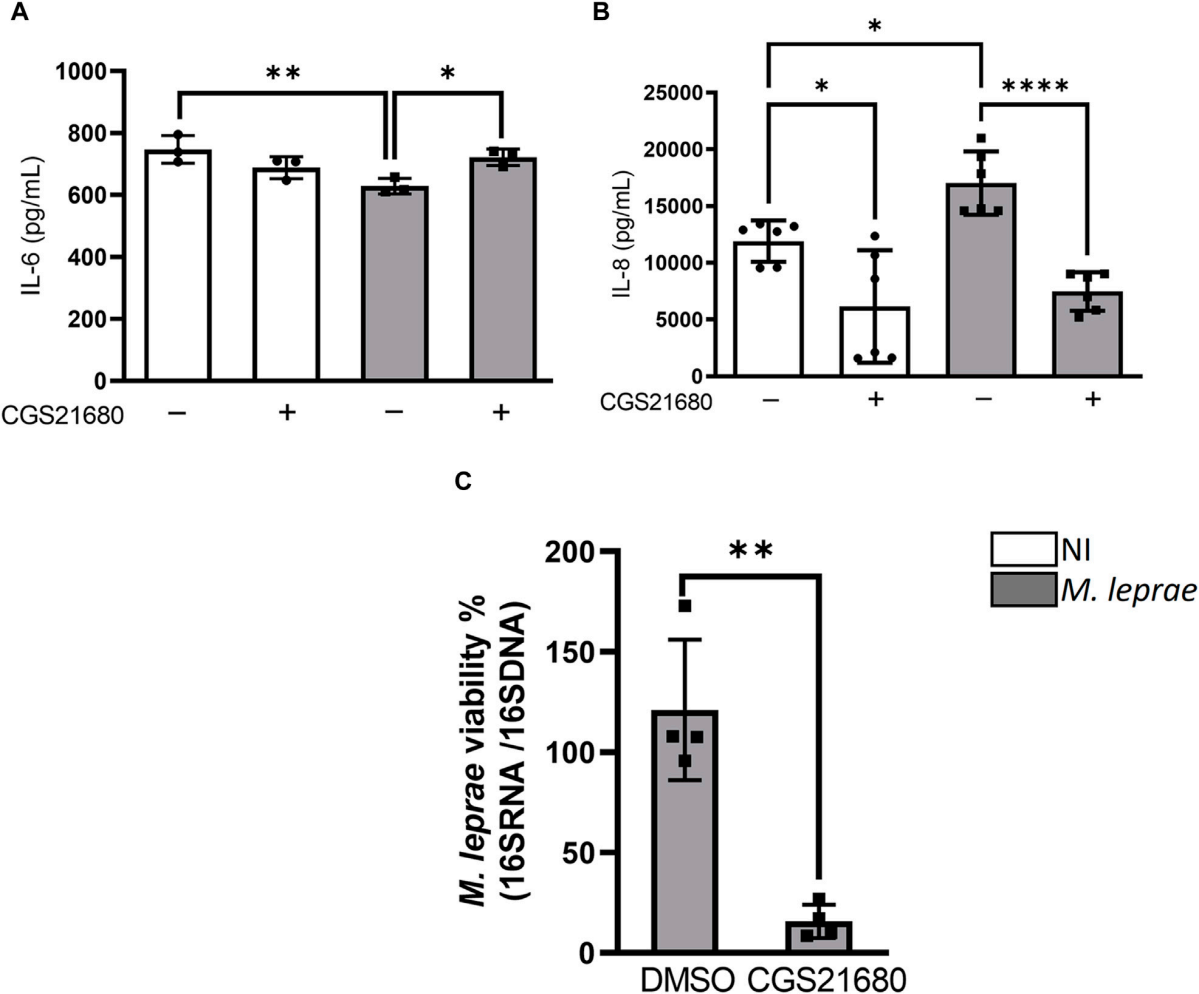
A_2A_R agonist treatment reverses production of IL-6 and IL-8 cytokines and decreases intracellular *Mycobacterium leprae* viability. **(A,B)** 4 × 10^4^ SCs treated (gray) or not (white) with A_2A_ agonists CGS2168 (100 µM) and infected with *M. leprae* (MOI 5:1) for 48 h. Supernatant collected and cytokines **(A)** IL-6 and **(B)** IL-8 were quantified by ELISA. Statistical differences evaluated via one-way ANOVA with Bonferroni’s multiple comparisons test **(C)**. *M. leprae* viability measured by RT-qPCR using ratio of 16S cDNA/16S DNA 48 h after infection of SCs pre-treated or not with 100 μM CGS21680. Differences between groups assessed using parametric Student’s t-test. *p < 0.05, **p < 0.01 and ****p < 0.0001.

**FIGURE 9:**
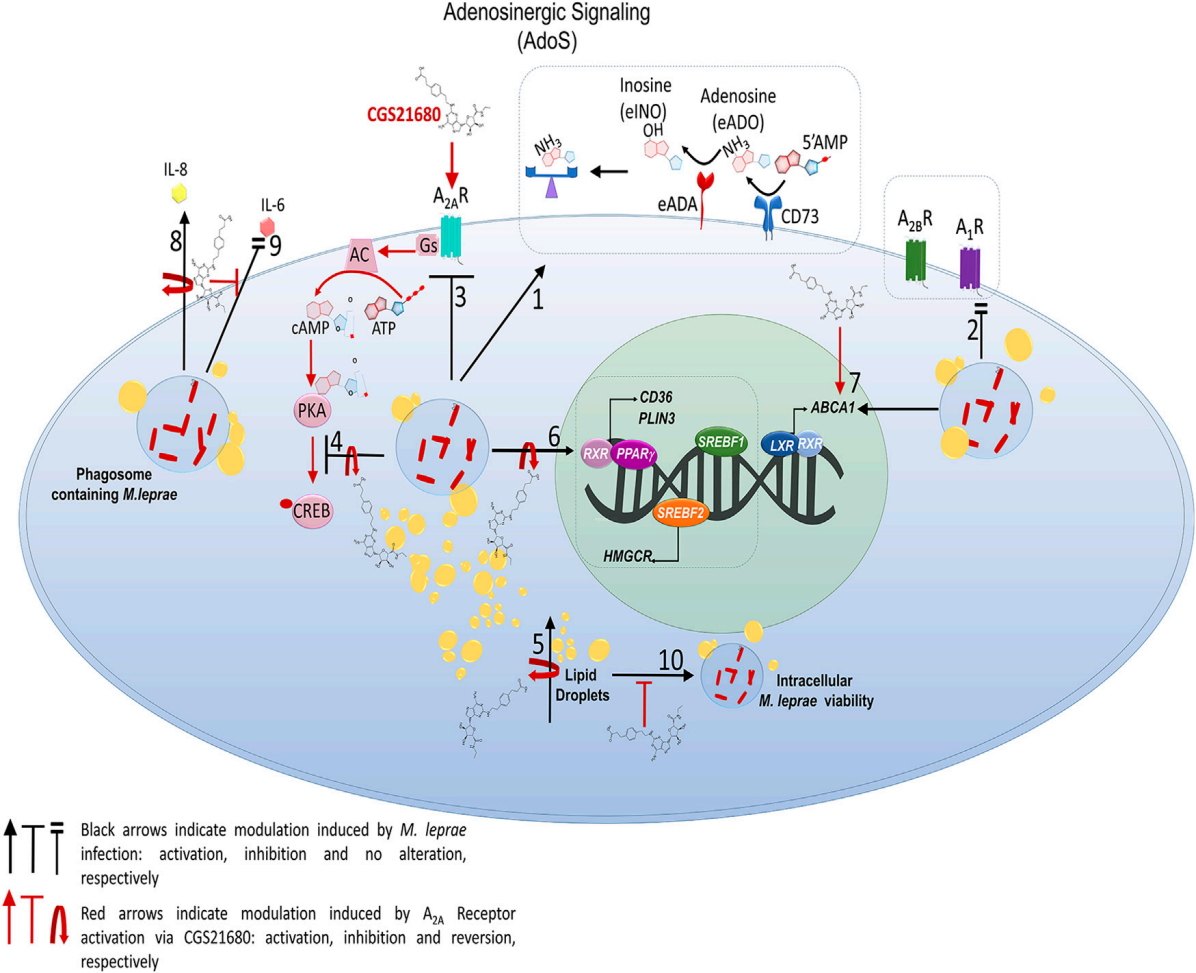
Schematic representation of impact of A_2A_R signaling on lipid metabolism in *Mycobacterium leprae-*infected Schwann cells 1. *M. leprae* increases the expression and activity of the enzyme CD73 and increases the expression of ADA, that regulation potentially decreasing the e-ADO level in the extracellular environment; 2. *M. leprae* does not affect expression of A_2B_ and A_1_ receptors; however, 3. *M. leprae* infection inhibits expression of A_2A_R and, 4. decreases phosphorylation of transcription factor CREB whose effect is reversed with CGS21680, the agonist of A_2A_R, 5. CGS21680 reverses LDs accumulation induced by *M. leprae* due: 6. downregulation of the expression of lipogenic genes such as *CD36, PLIN3, PPARy, SREBF1* and *HMGCR*, already known to be upregulated during *M. leprae* infection (Mattos et al., 2014). 7–8. A_2A_R when activated by CGS21680 upregulates gene *ABCA1* involved in cholesterol efflux. 9. CGS21680 inhibits IL-8 cytokine production induced by *M. leprae,* and 10. reduces the extracellular levels of IL-6 which were maintained at the level of control by *M. leprae infection*. Finally, 11. CGS21680 when activating A_2A_r potentially decreases intracellular *M. leprae* viability by reversing the molecular mechanisms essential for its maintenance and persistence in SCs, thus contributing to the establishment of the infection.

P1 receptors have been associated both with a pro-inflammatory response (Csóka et al., 2007) and pro-resolving and anti-inflammatory effects (Alam et al., 2009). IL-8 is a cytokine that is positively modulated by *M. leprae* infection, both in macrophages (Mattos et al., 2009) and Schwann cells (Souza et al., 2022), while IL-6 is downregulated in *M. leprae*-infected macrophages (Polycarpou et al., 2016). The literature has shown that A_2A_R regulates the production of both IL-6 and IL-8 in different study models (Köröskényi et al., 2016; Colombini et al., 2020). We wondered whether A_2A_R could affect the production of these cytokines modulated by *M. leprae* infection. Thus, SCs were infected with *M. leprae* and treated with CGS21680. The results revealed that *M. leprae* slightly decreased, with statistically significance, the extracellular levels of IL-6 and increased the production of IL-8—the latter already described in the literature (Díaz Acosta et al., 2018)—compared with the NI SCs (Figures 8A, B). Interestingly, the addition of CGS21680 increased the extracellular levels of IL-6 but inhibited the levels of IL-8 both in NI and *M. leprae*-infected SCs (Figures 8A, B) suggesting that the downregulation of A_2A_R in infected SCs contributes to the maintenance of the extracellular levels of both IL-6 and IL-8 produced by *M. leprae*-infected SCs. These statistical data are summarized in Supplementary Table S7. Finally, we assessed through RT-qPCR whether the activation of A_2A_R could impact *M.* leprae intracellular viability. We observed that the addition of CGS21680 significantly reduced *M. leprae* viability in comparison with untreated SCs (Figure 8C). Overall, the observed data suggests that *M. leprae*, by downmodulating the expression of A_2A_R, leads to decreased downstream signaling from this receptor, which contributes to the maintenance of the LD accumulation and intracellular viability of the bacilli. These results are schematically summarized in Figure 9.

## 4 Discussion

This work demonstrated for the first time the role of the AdoS—precisely, the CD73-ADA-A_2A_R axis—in the interaction of *M. leprae-*SCs. We showed that the ectoenzyme CD73 is upregulated whereas the ADA enzyme is downregulated in *M. leprae-*infected Schwann cells. These findings imply that the precise regulation of eADO levels is necessary. Furthermore, we show that ST88-14 SCs express the A_1_, A_2A_, and A_2B_ receptors; the infection only downmodulates A_2A_R, which is essential to maintaining the LDs accumulation and *M. leprae* intracellular viability.

AdoS acts as a target for many disorders in various models of inflammation and in neurodegenerative and infectious disease (Eckle et al., 2008; Visentin et al., 2013; Li et al., 2015; Eberhardt et al., 2022). Leprosy is a long-lasting infectious peripheral neuropathy that can manifest in various clinical forms and is directly linked to the patient’s immune response. As a result, researching the effect of AdoS on this disease could provide valuable insights into the pathogenesis, tolerance, and survival mechanisms triggered by *M. leprae* infection.

Here, we focused on the CD73-A_2A_R-ADA axis as a starting point for investigating the involvement of the purinergic system in leprosy pathogenesis. CD73 hydrolyzes extracellular 5′AMP to eADO, which, once outside of cells, can activate specific G protein-coupled receptors (Huang et al., 2021). In addition, eADO can be converted into eINO by ecto-ADA or taken back up by equilibrative nucleoside transporters (ENTs) (Alarcón et al., 2020). Here, we show that CD73 is upregulated in *M. leprae*-infected SCs. CD73 is a pivotal enzyme in eADO production and plays an essential role in maintaining equilibrium by converting eATP-induced immune stimulation into eADO-induced immunosuppression (Shao et al., 2023). Previous studies have demonstrated that CD73 is associated with bacterial escape, enhancing neutrophil recruitment during *Mycobacterium tuberculosis* infection (Dubois-Colas et al., 2014; Petit-Jentreau et al., 2015). CD73 is crucial for reducing tissue damage and controlling *Toxoplasma gondii* infection (Yilmaz et al., 2017), and it increases *L. amazonensis* survival via immunomodulatory effects activated by adenosine receptors (Basu et al., 2020; Bajracharya et al., 2022).

Although eADO is an immunosuppressor molecule, its effects—depending on the adenosine receptor—as a signaling molecule can be pro- or anti-inflammatory, and the enzyme ADA plays an essential role in this context (Bhagavatham et al., 2021). We showed that SCs infected with *M. leprae* increased ADA expression. Since previous studies demonstrated that ADA could lead to impaired immune functions, we can hypothesize that the bacilli may modulate it to maintain pathogen survival inside host cells. However, it is crucial to consider the possible effects of INO on leprosy due to its anti-inflammatory and antinociceptive properties (Nascimento et al., 2010; Nascimento et al., 2015).

Research in pharmacology and expression of receptors has revealed a wide range of purinergic receptors in all major glial classes, including SCs (Welihinda et al., 2016), suggesting an intercellular communication route to establish a functional connection between neurons and glia. Additionally, research has revealed the elevated expression of A_2A_R and A_2B_R in non-myelinating SCs (Fields and Burnstock, 2006; Patritti-Cram et al., 2021). According to Patritti-Cram et al. (2021), mouse SCs exhibit considerable expression of the A_1_R, A_2A_R, and A_2B_R but low expression of the A_3_R. Interestingly, the primary receptors involved in neuroinflammation modulation are A_1_R and A_2A_R (Martí Navia et al., 2020).

A_2A_R and A_2B_R interact with stimulatory GTP-binding protein (Gs), resulting in increased intracellular cAMP levels that activate the cAMP-dependent protein kinase (PKA), which phosphorylates cAMP response element-binding protein (CREB) at Ser133 (p-CREB) (Darashchonak et al., 2014; Yang et al., 2015). p-CREB regulates the transcription of several genes related to various physiological conditions, such as cell immune responses, lipid metabolism, cellular survival, proliferation, and differentiation (Wen et al., 2010; Darashchonak et al., 2014; Yang et al., 2015). Plenty of genes involved in the immune system contain this cAMP-responsive element, including IL-6, IL-10, and TNF-α coding genes (Wen et al., 2010). Additionally, p-CREB suppresses the NF-κB, controlling the inflammatory responses by reducing IL-8, which contains functional binding sites for NF-kB in its promoter region (Shaywitz and Greenberg, 1999; Wen et al., 2010; Bezzerri et al., 2011; Iizuka et al., 2020).

Our data have shown that *M. leprae-*infected SCs downregulate the expression of A_2A_R; additionally, the p-CREB level is significantly reduced compared to non-infected cells. Nevertheless, adding a specific A_2A_R agonist increased p-CREB expression in non-infected cells and reversed the effect of *M. leprae* in the infected SCs, showing that A_2A_R is functional. Recently, CREB was shown to play a role in *M. tuberculosis*-infected human macrophages (Leopold Wager et al., 2023). In this study, *M. tuberculosis-*induced p-CREB was independent of cAMP but dependent on the p38/MAPK pathway. In our model, *M. leprae* downregulates the levels of p-CREB, which reinforces CREB involvement in mycobacterial infections.

The canonical pathway activated by A_2A_R is known to involve the cAMP-PKA-CREB axis (Fields and Burnstock, 2006). Nevertheless, CREB can be phosphorylated by alternative enzymes such as protein kinase C (PKC), pp90 ribosomal S6 kinase, calmodulin kinases, and p38-MAPK (Brindle et al., 1993; Wong and Sakamoto, 1995; Shaywitz and Greenberg, 1999; Gubina et al., 2001; Gee et al., 2006; Gee et al., 2007). Therefore, understanding how *M. leprae* downregulates A_2A_R and how this receptor is connected to the CREB pathway will reveal new insights into their role in leprosy pathogenesis.

The impact of A_2A_R in producing two cytokines by SCs, IL-6, and IL-8 was also investigated. The results have shown that *M. leprae* lowers the extracellular levels of IL-6, which supports previous research on infected macrophages (Csóka et al., 2007; Mattos et al., 2009; Mattos et al., 2014) but that it increases IL-8 secretion, as also shown by Díaz Acosta et al. (2018). However, CGS21680 treatment has reversed both effects caused by *M. leprae* infection. These findings suggest that the decreased expression and activity of A_2A_R participate in the maintenance of low levels of IL-6 and high levels of IL-8. Even though the involvement of CREB in this response induced by CGS21680 has yet to be studied, we could suggest that increasing p-CREB could elevate IL-6 through binding to the IL-6 promoter region (Wen et al., 2010). On the other hand, the inhibition of IL-8 production may result from the suppression of NF-kB, which regulates IL-8 transcription. An elevated level of IL-8 in the infected cell has been considered a possible neuroinflammatory mechanism triggered by *M. leprae*, which can be involved in the demyelinating phenotype in leprosy neural injury (Díaz Acosta et al., 2018). The literature indicates that A_2A_R has a neuroprotective and promyelinating role (Melani et al., 2006; Shen et al., 2018). Therefore, our findings may open new perspectives into the role of A_2A_R in leprosy; perhaps the downregulation of A_2A_R is a mechanism used by *M. leprae* that may contribute to leprosy neural injury?

Recently, several studies have focused on the role of A_2A_R in the atherosclerotic process, which involves both lipid accumulation and the activation of inflammatory pathways (Voloshyna et al., 2013). ARs regulate cellular cholesterol homeostasis by modulating intracellular cholesterol trafficking. Niemann–Pick type C1 (NPC1) is a membrane protein expressed on endosomes and lysosomes that mediates intracellular cholesterol trafficking. A_2A_R agonist CGS 21680 significantly decreases intracellular cholesterol accumulation and increases lysosomal calcium and subsequent mitochondrial function in a fibroblast cell line derived from NPC1 patients. The beneficial effects of A_2A_R agonists were blocked by the A_2A_R antagonist ZM241385, suggesting that this activity is dependent on the activation of A_2A_R (Colombini et al., 2020).

The concentration of CGS21680 chosen for this study was based on data from the literature, including Yasuda et al. (2003), showing a potency rank order for A_2B_R in hepatocytes using 100 μM NECA (N-ethylcarboxamidoadenosine), a non-selective agonist of adenosine receptors, as well as adenosine and CGS21680. They describe a potency rank of NECA > adenosine > CGS21680 in the accumulation of cAMP and stimulation of glycogenolysis and gluconeogenesis in rat hepatocytes. Another study that gave us support to test CGS21680 at a concentration of 100 μM was Lebon et al. (2015), where the molecular determinants of the human A_2A_R were analyzed using a thermostabilized construction of the conformational structure of the A_2A_R, bound to the agonist CGS21680 at a concentration of 100μM. This revealed the high selectivity of CGS21680 for A_2A_R compared to A_2B_R. Their results also showed that differences in the amino acid sequence of the extracellular loop 2 of these receptors were responsible for this phenotype (Lebon et al., 2015). Our findings indicate that *M. leprae* infection significantly decreases the expression and activity of A_2A_R, as measured by CREB phosphorylation. Additionally, we observed that the A_2B_ receptor is more abundant in *M. leprae*-infected SCs than A_2A_R, as confirmed by RT-qPCR and immunofluorescence analysis. Based on these results, we decided to use a high concentration of both adenosine and CGS21680 to test our hypothesis that the downregulation of A_2A_R by *M. leprae* may be important for infection to be established.

We showed that 100 µM extracellular adenosine reversed the formation of *M. leprae*-induced LDs in SCs. Since A_2A_R is described as an anti-lipogenic receptor, we investigated whether *M. leprae* reduces the expression and activity of A_2A_R as a protective mechanism that assists in the establishment of infection, the maintenance of high levels of CLs and ultimately, the viability of the bacillus. For this, we maintained the excess from CGS21680 (100 μM) to assess nonspecific binding, and we used specific antagonists for A_2A_R and A_2B_R. The results confirmed that the LD’s reduction was related to the A_2A_R and not to A_2B_R. To investigate whether lipogenic molecular mechanisms were downregulated or upregulated by A_2A_R activation in *M. leprae*-infected SCs, RT-qPCR analysis was carried out. One of the genes analyzed in our study was *PPARγ,* which encodes a key transcription activator that regulates several lipid metabolism genes, including *CD36* and *PLIN3,* which respectively encode a scavenger receptor for oxidized low-density lipoprotein and LD-associated proteins from perilipin family (Shi et al., 2013; Ferrante et al., 2016). CD36 was already observed by RT-qPCR and Western blotting analysis (Mattos et al., 2014). Recently, we showed that *M. leprae*-infected SCs display increased uptake of LDL-cholesterol (Maréchal et al., 2018). Here, we show that *M. leprae* increases the expression of *CD36*, which may contribute to the LDL-cholesterol uptake previously observed (De Nuccio et al., 2019), and that treatment with CGS21680 reverses this effect. This result suggests that the downregulation of A_2A_R can also be a mechanism contributing to the uptake of exogenous cholesterol by *M. leprae* infection. The effect of A_2A_R on CD36 downregulation has already been described (Desai et al., 2005), corroborating the data obtained in this work. Furthermore, we observed an upregulation of *PLIN3* by infection and a downregulation by CGS21680.

We investigated the impact on *PPARγ* gene expression. As already described, SCs infected by *M. leprae* increase the expression of PPARγ (Mattos et al., 2014; Rosa et al., 2021); however, we show that CGS21680 treatment reverses this effect. Based on these results, we suggested that the lower level of LD in infected SCs treated with CGS21680 may be due to the downregulation of *PPARγ* and *CD36*, which affects *PLIN3*. Interestingly, feedback between PPARγ and A_2A_R has already been described in lung damage in acute lung injury (Díaz Acosta et al., 2018) and a vascular response model (He et al., 2013). Therefore, exploring the PPARγ -A_2A_R axis in leprosy may provide relevant information about this disease.

It is known that inflammation, a process usually inhibited by adenosine (mainly by action on A_2A_R and A_2B_R), is an essential factor in promoting foam cell formation and atherosclerosis (Koupenova et al., 2012; Nayeem et al., 2013). Here, we investigated two inflammatory cytokines modulated by *M. leprae* infection (Mattos et al., 2014): IL-6 and IL-8. Johnston-Cox et al. (2012) showed the involvement of IL-8 in hyperlipidemia and coronary disease. We already showed a cross-talk between the cytokine IL-8 and PPARγ (Díaz Acosta et al., 2018), and this cross-talk had previously been described in *M. tuberculosis-*infected macrophages (Shen et al., 2018). We confirmed that *M. leprae* increased IL-8 production but that CGS21680 treatment decreased this effect, which may suggest that A_2A_R could down-modulate IL-8 and, consequently, PPARγ, or *vice versa*. However, further research is necessary to determine the actual impact of this cross-talk on LD accumulation and how A_2A_R affects this axis.

The *SREBF1* and *SREBF2* genes were also included in this analysis, and both were already identified as upregulated in skin biopsies of multibacillary (MB) patients compared to PB (Mattos et al., 2014). The proteins encoded by these genes, SREBP1 and SREBP2, regulate fatty acid and *de novo* cholesterol biosynthesis, respectively. In this same work, a gene regulated by *SREBF2*, such as 3-hydroxy-3-methyl-glutaryl-coenzyme A reductase (*HMGCR*) that encodes the enzyme HMGCR that catalyzes the rate-limiting step in cholesterol biosynthesis, was also upregulated in an LL patient (Mattos et al., 2014). Our work showed that *M. leprae* upregulated *SREBF1* in infected SCs and that CGS21680 inhibited this phenotype. The *SREBF2,* which regulates *de novo* cholesterol synthesis genes, was not modulated either by *M. leprae* infection or A_2A_R activation. However, the gene *HMGCR,* whose transcription is regulated by *SREBF2,* was upregulated by *M. leprae* infection, and the A_2A_R activation inhibited this effect. This suggests that despite gene expression not being altered, *SREBF1* is functional.

Besides the genes involved in lipid synthesis, we also investigated the impact of CGS21680 on the genes involved in cholesterol efflux, such as *ABCA1* and *CYP27A1.* The results obtained here corroborate previous data from the literature, where activation of A_2A_R increased cholesterol efflux by enhancing the expression of the reverse cholesterol transporter *ABCA1*. Nonetheless, although *M. leprae* infection increased the expression of *CYP27A1* in our study, CGS21680 did not reverse it. It is essential to highlight that *ABCA1* is regulated by the transcription factor LXR, which regulates the transcription of other genes such as *CH25H* (Dechkhajorn et al., 2020), which encodes cholesterol 25-hydroxylase (CH25H) that produces 25-OH-cholesterol (25-HC), another LXR activator (Akiyama et al., 2002; Liu et al., 2018; Matsuo, 2022). Several studies have investigated the regulatory mechanisms of CH25H expression. It was observed that 25-HC could activate its expression, forming a positive feedback loop, and this effect was dependent on LXR activation [18]. Furthermore, inflammatory cytokines such as IL-6 can promote CH25H expression (Magoro et al., 2019). Therefore, other enzymes from the CYP450 family, such as CH25H, could further activate LXR via A_2A_R, increasing the transcription of *ABCA1*. For example, the increase in IL-6 induced by CGS21680 could be one of these factors.

Therefore, our study model suggests that A_2A_R decreased LD accumulation by preventing lipogenesis and inducing cholesterol efflux in infected SCs. Nevertheless, we must not discard the importance of both A_1_R and A_2B_R in *M. leprae* infection since the literature shows they both control lipolysis and lipogenesis (Johansson et al., 2008; Koupenova and Ravid, 2013; Leiva et al., 2017).

Many studies have demonstrated the relevance of the purinergic system during various infectious, inflammatory, and neurodegenerative diseases, focusing on the P2-receptors—mainly P2X7 (Jacob et al., 2013; Di Virgilio et al., 2017). Souza et al. (2021) recently reported that the P2X7 receptor may exert a vital role in the death of *M. leprae*, which is necessary to control the disease. Consequently, the significance of investigating the influence of the purinergic signaling through P2 receptors in leprosy is also mandatory, primarily because the equilibrium between eATP and eADO is crucial for avoiding unregulated tissue harm caused by exacerbated inflammatory reactions. This duality in the purinergic system in a complex disease such as leprosy, which presents itself in diverse clinical manifestations primarily because of the host’s immune reaction, might provide extremely significant perspectives on its progression.

## 5 Conclusion

The evidence presented here shows that the activation of A_2A_R reverses both the host cell lipid metabolism subverted by *M. leprae* in infected SCs and the levels of IL-6 and IL-8 produced by infected SCs; this may be involved directly in the effects produced by A_2A_R activation on the LD accumulation. Overall, our results show for the first time that the modulation of the CD73-ADA-A_2A_ axis by *M. leprae* infection helps maintain a functional strategy to ensure bacilli survival in SCs. Our findings also present innovative data introducing new perspectives for exploring leprosy neuropathogenesis and can offer new targets to develop complementary therapies for the treatment of this disease.

## Data Availability

The original contributions presented in the study are included in the article/Supplementary Material; further inquiries can be directed to the corresponding author.

## Supplementary material

SUPPLEMENTARY FIGURE S1 CGS21680 50 nM decreases *M. leprae*-induced lipid droplets accumulation in Schwann cells. 4 × 10^4^ SCs were infected with *M. leprae* (MOI 5:1) for 48 h, and LDs were visualized by fluorescence microscopy using Oil Red O (ORO) staining. The quantification of ORO-stained LDs was plotted as measurement of LD area/cell. A total of 100 cells for each condition was analyzed. **(A)** The pretreatment with 50 nM CGS21680 reduced the LD formation induced by *M. leprae*, and the specific A_2A_R antagonist ZM241385 (1 µM) reversed the inhibitory effect of CGS21680 in *M. leprae* induced LD. Scale bar 10 μm (white line). **(B)** LDs quantification by Image J software. NI= not infected. Data are shown as mean± SD of at least three different experiments performed in triplicate. Statistics used the NI condition as a reference. Data were evaluated via parametric student t-test, **p* < 0.05, **p* < 0.01.

SUPPLEMENTARY FIGURE S2 A_2B_ receptor inhibition does not affect the inhibitory effect of CGS21680 on lipid droplet formation in *M. leprae*-infected Schwann cells. 4 × 10^4^ SCs were infected with *M. leprae* (MOI 5:1) for 48 h. LDs were visualized by fluorescence microscopy using Oil Red O (ORO) staining. The quantification of ORO-stained LDs was plotted as measurement of LD area/cell for a total of 200 cells for each condition. **(A,B)** The use of the specific A_2B_R antagonist MRS1754 (1 µM) does not *per se* affect the formation of LDs and does not reverse the effect of CGS21680 (100 µM) in inhibiting LD induced by *M. leprae* infection. Scale bar 20 μm (white line). **(B)** LDs quantification by Image J software. NI= not infected. NT= not treated. Data are shown as mean± SD of at least three different experiments performed in triplicate. Statistical differences were evaluated via one-way ANOVA with Bonferroni’s multiple comparisons test, **p* < 0.05, ***p* < 0.01, and ****p* < 0.0001.

SUPPLEMENTARY FIGURE S3 A_2B_ receptor expression is not modulated by *M. leprae*-infected Schwann cells. 4 × 10^4^ SCs were infected with *M. leprae* (MOI 5:1) for 48 h. The expression of the A_2B_ receptor was analyzed. **(A)** Protein expression of A_2B_R (red) was visualized using immunofluorescence. DAPI nuclear staining in blue. Scale bar: 20 µm. **(B)** Mean fluorescence intensity (MFI) quantified using ImageJ software. NI= not infected. All data are mean ± SD. Statistical significance was determined using Student parametric t-test.

SUPPLEMENTARY FIGURE S4 Schematic representation of an interactome between the adenosinergic proteins and of the lipid homeostasis analyzed in *M. leprae*-infected Schwann cells The interactome represents all the proteins or genes analyzed in this work: *ADORA2A, NT5E, ADA, IL-6; IL-8 (CXCL8) PLIN3; PPARy; CD36; SREBPF1; SREPBF2; HMGCR; ABCA1; CYP27A1*. Interactome was built using network STRING version 12.0 (https://string-db.org/). Each node represents the proteins with predicted 3D structures presented within the nodes. **(A)** Interactome showing in gray lines the degree of confidence of association defined by their thickness, indicating the strength of data support. Green and red nodes represent MCL clustering of these proteins. **(B)** Interactome of each cluster presented in Figure A. The color lines show how these proteins are connected: blue lines indicate curated database, purple lines were experimentally determined, yellow lines indicate information from text mining, black lines indicate co-expression, dark blue gene co-occurrence, and light blue indicates protein homology. The color inside the nodes represents the associated cellular processes based on gene ontology.
