# Supplementary figures and images for: Adenosine A2A receptor as a potential regulator of Mycobacterium leprae survival mechanisms: new insights into leprosy neural damage
Source: Front Pharmacol. 2024 Jun 28;15:1399363. doi: 10.3389/fphar.2024.1399363 (PMC11239521; doi:10.3389/fphar.2024.1399363)

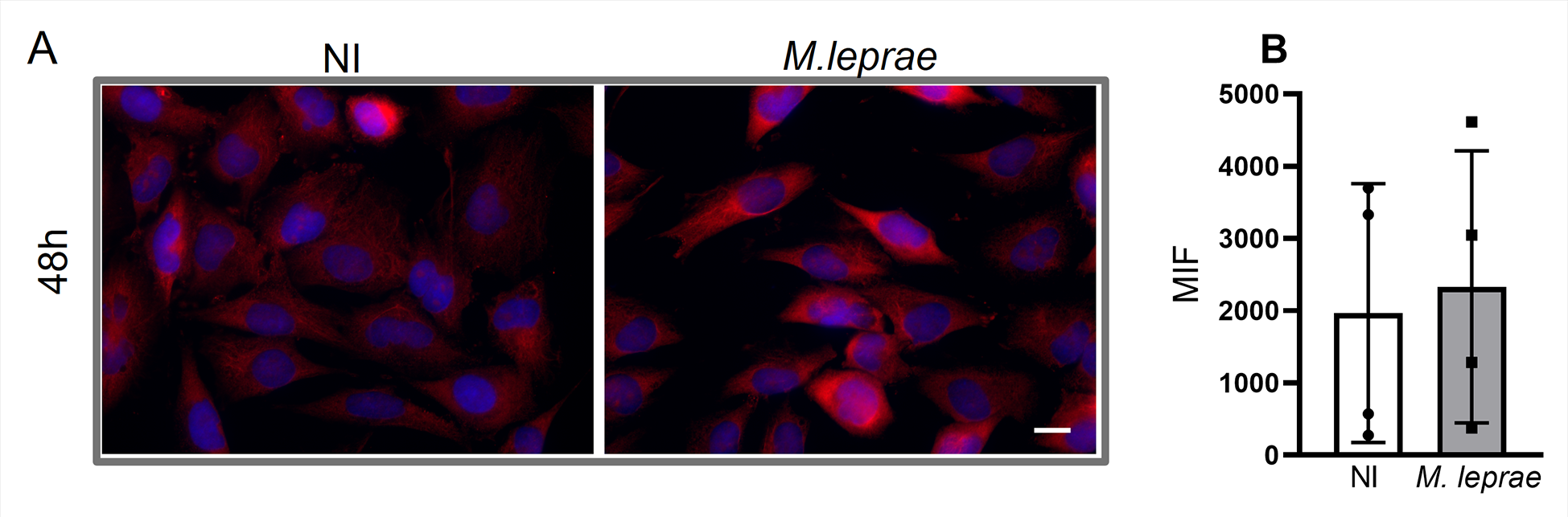

Supplement: Supplementary file 3 [file Image3.TIF]

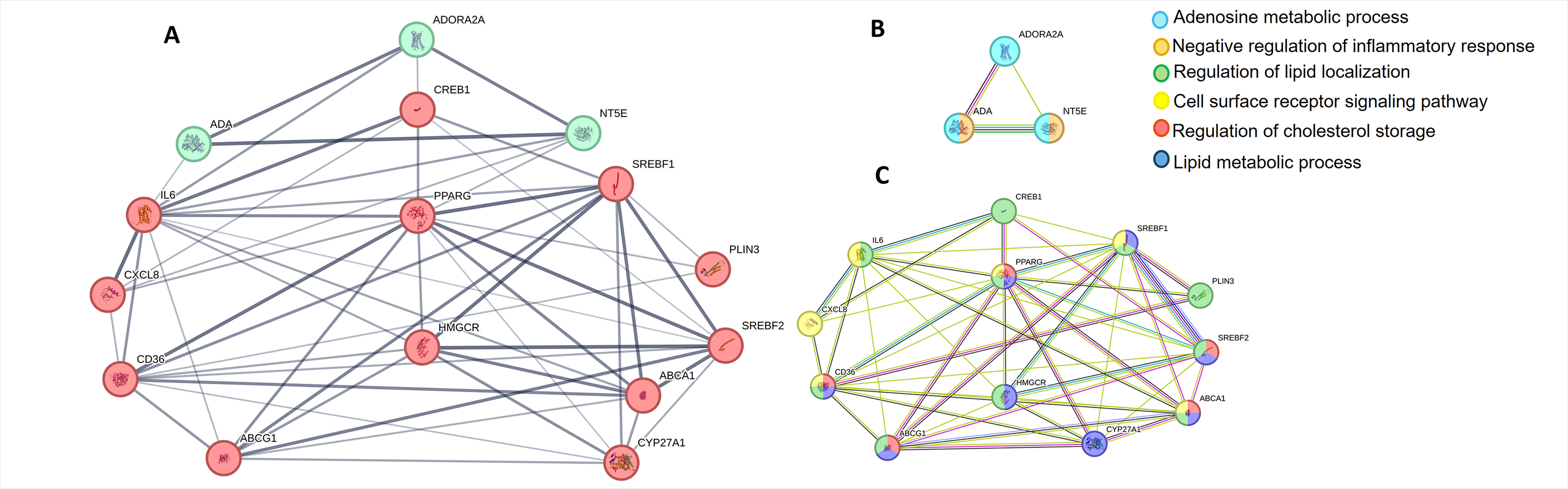

Supplement: Supplementary file 4 [file Image4.TIF]

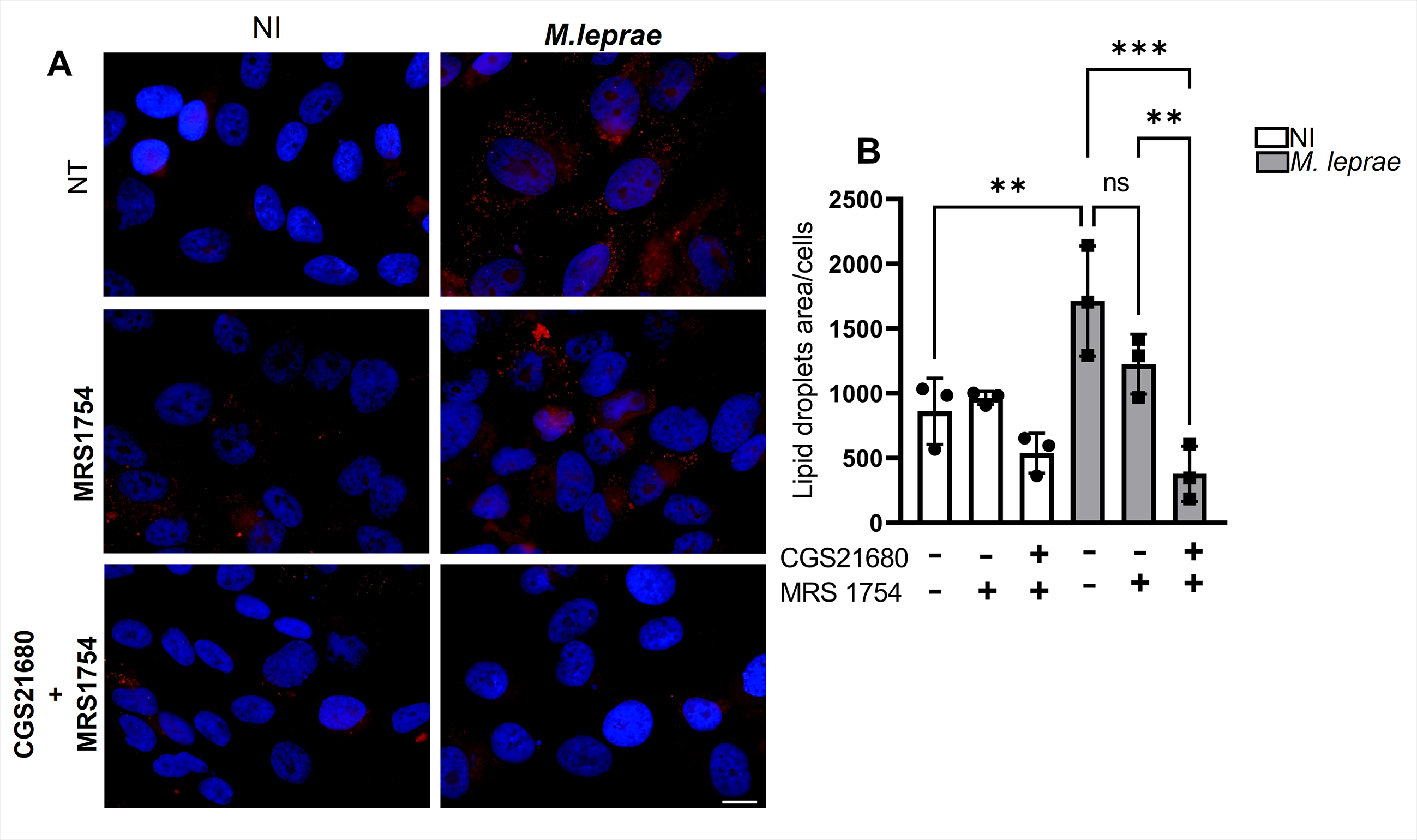

Supplement: Supplementary file 5 [file Image2.TIF]

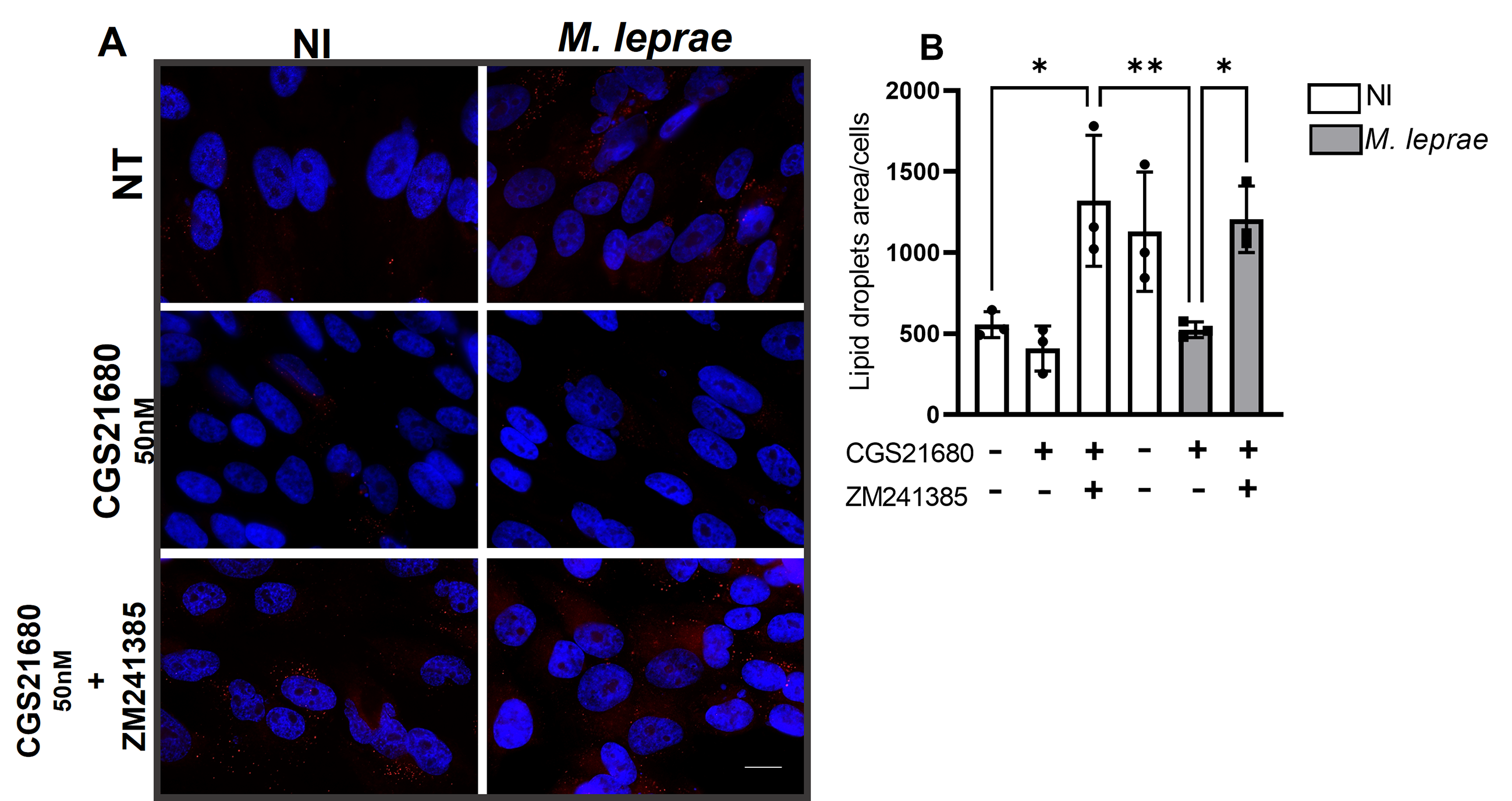

Supplement: Supplementary file 6 [file Image1.TIF]
